# A meta-analysis of crop response patterns to nitrogen limitation for improved model representation

**DOI:** 10.1371/journal.pone.0223508

**Published:** 2019-10-17

**Authors:** Verena Seufert, Gustaf Granath, Christoph Müller

**Affiliations:** 1 Liu Institute for Global Issues and Institute for Resources, Environment and Sustainability (IRES), University of British Columbia, Vancouver, Canada; 2 Department of Ecology and Genetics, Evolutionary Biology Centre, Uppsala University, Uppsala, Sweden; 3 Potsdam Institute for Climate Impact Research (PIK), Member of the Leibniz Association, Potsdam, Germany; Tennessee State University, UNITED STATES

## Abstract

The representation of carbon-nitrogen (N) interactions in global models of the natural or managed land surface remains an important knowledge gap. To improve global process-based models we require a better understanding of how N limitation affects photosynthesis and plant growth. Here we present the findings of a meta-analysis to quantitatively assess the impact of N limitation on source (photosynthate production) versus sink (photosynthate use) activity, based on 77 highly controlled experimental N availability studies on 11 crop species. Using meta-regressions, we find that it can be insufficient to represent N limitation in models merely as inhibiting carbon assimilation, because in crops complete N limitation more strongly influences leaf area expansion (-50%) than photosynthesis (-34%), while leaf starch is accumulating (+83%). Our analysis thus offers support for the hypothesis of sink limitation of photosynthesis and encourages the exploration of more sink-driven crop modelling approaches. We also show that leaf N concentration changes with N availability and that the allocation of N to Rubisco is reduced more strongly compared to other photosynthetic proteins at low N availability. Furthermore, our results suggest that different crop species show generally similar response patterns to N limitation, with the exception of leguminous crops, which respond differently. Our meta-analysis offers lessons for the improved depiction of N limitation in global terrestrial ecosystem models, as well as highlights knowledge gaps that need to be filled by future experimental studies on crop N limitation response.

## Introduction

Most terrestrial primary productivity is limited by nitrogen (N) availability, as N is rather costly to obtain in a form that is useable by plants [1,2]. Under future carbon-dioxide (CO_2_) fertilization, limitations of net primary productivity by N availability might become even more pronounced [3–5]. Currently the representation of N processes remains a key uncertainty both in global models of the natural [5–7], as well as of the managed [8,9] land surface. To allow better representation of N processes and better project the future terrestrial carbon (C) sink, as well as food production under a changing climate, we need to improve our understanding of the physiological processes underlying plant N limitation.

Theoretically, under N limitation plants can follow two strategies: either reducing N-allocation to leaves (i.e. the leaf N content, N_L_) and consequently their photosynthetic rate, or maintaining N_L_ rather constant and reducing leaf growth and leaf area [10,11]. To what degree plants adapt N_L_ under low N supply or keep N_L_ constant and adapt leaf area is still an on-going debate. Grindlay [10] hypothesized that under a range of N availability that is typical for field conditions, plants generally do not change their N_L_ significantly, but rather respond to N limitation mainly through a reduction in leaf area development. Others [12,13] have suggested different response patterns in different crop types (e.g. C3 vs. C4 or mono- vs. dicotyledonous species), and yet others conclude that there are consistent differences in responses between crop species but that these differences cannot be related to different crop types [11].

Under a limiting N supply, the question is whether N limitation primarily acts as a source (i.e. limiting the production of carbohydrates through photosynthesis) or as a sink limitation (i.e. limiting the use of carbohydrates for growth). One the one hand, N controls photosynthetic activity due to the dependence of photosynthetic rate on photosynthetic proteins (between 50 and 80% of total N_L_ is allocated to photosynthetic proteins in C3 plants; [14,15]). On the other hand, N also controls plant and leaf growth due to the dependence of new tissue development on enzymes, as well as structural proteins. Some evidence suggests that under N limitation reductions in leaf elongation rates precede and/or exceed reductions in photosynthetic rates [16–18], suggesting that sink limitation might be more important than source limitation. Beside attempts based on the concept of Nitrogen Nutrition Index (NNI, [19]) no study has been able to assess the relative importance of sink versus source limitation across experiments or species [20].

N limitation represented in global models of the terrestrial biosphere (denoted here as Terrestrial Ecosystem Models, TEMs, here including both models of the natural and managed vegetation) is typically implemented as a reduction in carbon assimilation [21–26]; only in few models it also directly influences leaf area [27,28], or plant growth rates [29]. If plants do, however, primarily reduce leaf area rather than photosynthesis and if N limitation acts more as a sink limitation than as a source limitation, models that depict N limitation as a limitation of photosynthesis would be implementing plant C-N interactions incorrectly [20], which could provide an explanation for the current inability of TEMs to replicate experimental data well [30].

With this general lack of understanding it remains unclear in what detail and in what form N limitation needs to be implemented in TEMs. A wealth of experimental studies on C-N interactions have, however, been conducted, but the evidence from these studies has not been synthesized to date. While meta-analyses examining the response of plant growth and photosynthesis to atmospheric CO_2_ ([CO_2_]) enrichment have been conducted widely and have delivered valuable insights into plant physiology under elevated [CO_2_] that have been useful for the development of TEMs [31–36], the influence of N limitation on plant physiology has not been evaluated to the same extent. Previous meta-analyses on C-N interactions have examined the response of soil processes [37–41] and terrestrial plant biomass [41–44] to N enrichment, as well as the global distribution of N limitation of primary productivity [2,45]. Here we conduct a meta-analysis of the response of different leaf-level variables to N limitation in crops, analysing interactions with the degree of N limitation, CO_2_ fertilization, and species identity (e.g. N fixation, taxonomic group). We particularly aim to provide insights into the question whether crop growth is primarily source- or sink limited under limited N availability, and whether different crop species and crop types differ in their response patterns in this regard.

We focus our study on crop species for two main reasons. First, crop models are increasingly applied at continental to global scales [46], but generalizable global parameters for crops are often still missing, as global databases of plant physiological parameters are typically focused on natural vegetation [47–49]. Secondly, crop species have been studied extensively under semi-controlled conditions and insights from crop studies have often provided important advances in plant physiology [50–52]. For a meta-analysis of the impacts of N limitation on photosynthesis and growth, crops thus represent useful model organisms.

## Materials and methods

### Data collection

The meta-analysis was restricted to laboratory or pot experiments, as N limitation is more clearly expressed under these semi-controlled conditions and in the physically restricted rhizosphere. We thus excluded field studies, as the N supply in the field is more difficult to control, high loss of N from the system can occur and field studies often do not report sufficient information on soil properties to assess the actual N supply to crops. Even in the majority of laboratory or pot experiments, a true control of the N supply (comparable to the control of temperature or light conditions) is not achieved [53,54], hence we refer to these experiments as semi-controlled.

**Fig 1:**
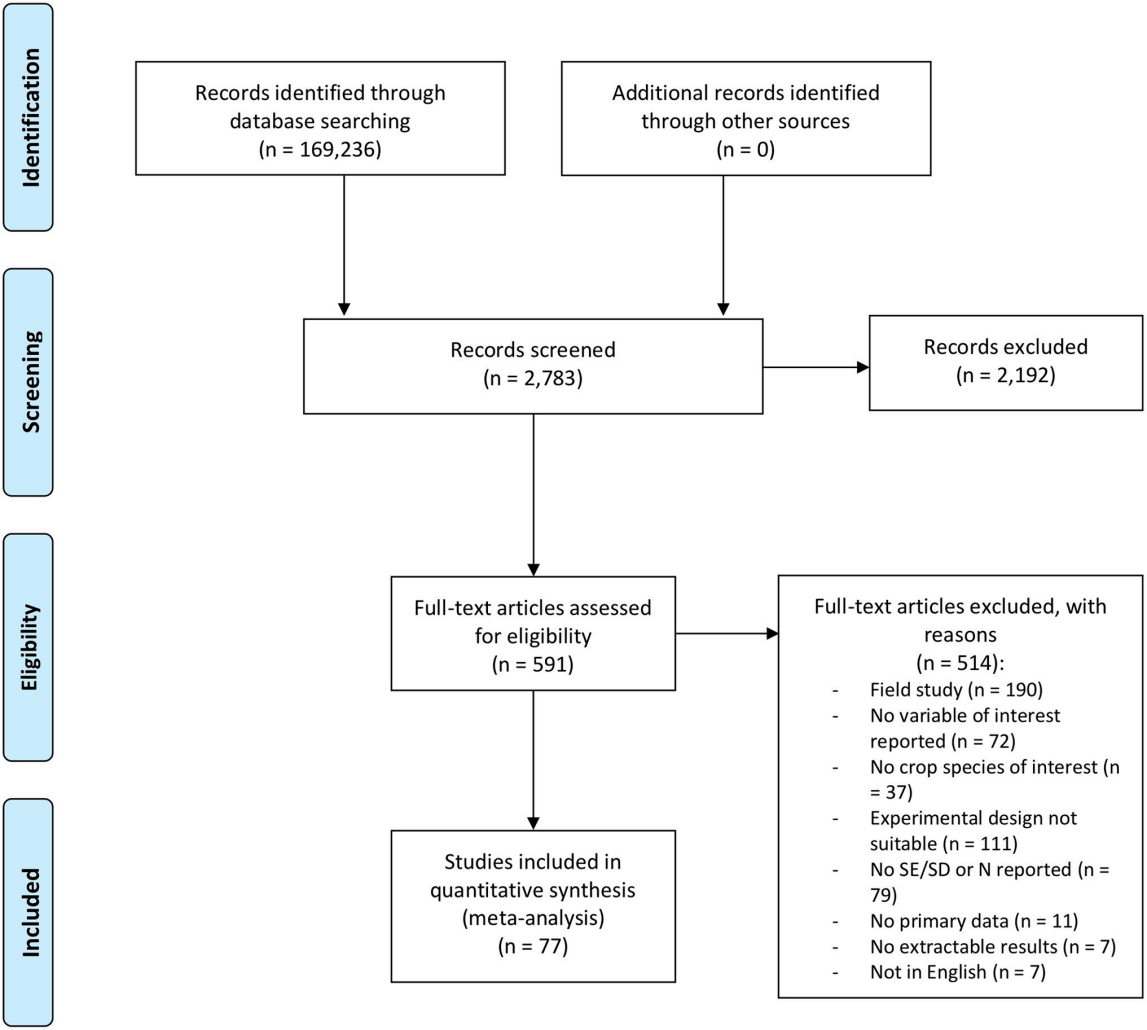
PRISMA flow diagram of database searching and article screening. The complete PRISMA checklist can be found in S2 Table.

A literature search was conducted for experimental studies published in peer-reviewed journals on crop photosynthesis under varying N supplies, using web-based search engines (Google Scholar and PubMed; search terms used were ‘nitrogen’ AND ‘photosynthesis’ AND ‘experiment’ combined with any of the crop species of interest; last search was conducted in November 2017) and by searching the reference lists of published articles. Many studies suitable for our analysis did not have the impact of N limitation on crop photosynthesis as their primary focus, but N availability was either varied only as a secondary experimental factor (e.g. in studies interested in the effects of elevated [CO_2_] on plant growth), and/or a photosynthetic variable of interest (e.g. leaf N content) was measured as part of a study interested in a different physiological outcome (e.g. nitrate transport in roots). We therefore had to use very general search terms in the literature search and the keywords used for searching literature databases yielded 169,236 records (Fig 1). Given that it was not logistically feasible to screen so many studies, and also given that the purpose of this meta-analysis was not to comprehensively summarize a clearly-delineated body of scientific literature, but rather to identify robust and generalizable patterns for better representation of N limitation effects on crop photosynthesis in TEMs, we cut-off the Google Scholar search results that we screened at pre-defined points, depending on the number of search records identified. For crop species with <2000 records identified in Google Scholar (i.e. *Pennisetum glaucum*, *Elaeis guineensis*, *Saccharum officinarum*, *Manihot esculenta*), we only screened the first 100 records (sorted by relevance); for crop species with > = 2000–10,000 records identified (i.e. *Gossypium hirsutum*, *Sorghum bicolor*, *Beta vulgaris*) we screened the first 140 records; and for crop species with >10,000 records identified (i.e. *Brassica napus*, *Glycine max*, *Hordeum vulgare*, *Oryza sativa*, *Phaseolus vulgaris*, *Solanum tuberosum*, *Triticum aestivum*, *Zea mays*) we screened the first 240 records. To decide how many search records to include in the screening, we chose a cut-off point that appeared feasible in terms of number of records to be screened, and then checked whether we could find additional suitable studies after our cut-off point for 3 crop species (i.e. *Triticum aestivum*, *Brassica napus*, *Manihot esculenta*). As no additional suitable studies could be found for these 3 crop species after our cut-off point, our study sample would most likely not have looked very different if we had screened more studies. This resulted in a total of 2740 records being screened (including all records identified through PubMed) (Fig 1), which—depending on crop species—represented 1–10% of all search results, and 591 full-text articles assessed for eligibility (Fig 1).

To be included in the analysis, studies had to meet the following criteria: (i) the study organism was a crop species of interest (S1 Table); (ii) at least one response variable of interest (Table 1) was measured; (iii) the study was conducted under semi-controlled conditions in laboratory or pot experiments; (iv) experiments were conducted with at least two different N supply rates; and (v) the mean (X), an error term (SD or SE) and sample size (n) for the response variable under different N supplies were reported as numerical or graphical data. From our search we found 77 articles that fulfilled our criteria (Fig 1; S3 Table). No review protocol was used or published for this meta-analysis.

**Table 1:** List of response variables examined in the meta-analysis.

Parameter	Definition
photosynthesis	leaf photosynthetic rate, measured at experimental [CO_2_] and either experimental or saturated light conditions (per unit area)
leaf area	leaf area (per plant or per leaf)
N_L_ per unit area	leaf N content per unit area
N_L_ per unit mass	leaf N content per unit dry weight
chlorophyll	leaf chlorophyll content (per unit area or per unit fresh weight)
Rubisco	Rubisco content (per unit area or per unit fresh weight)
SLA	Specific leaf area (leaf area per leaf dry weight)
leaf sugar	leaf sugar content (per unit area or per unit dry/fresh weight)
leaf starch	leaf starch content (per unit area or per unit fresh weight)

Additional leaf-level variables (e.g. leaf nitrate or amino acid contents, Rubisco activity, leaf protein content, total leaf non-structural carbohydrates or stomatal conductance) were not included due to the limited number of studies reporting such data. For several variables, we have combined different units to increase small sample sizes but tested for differences between these different units: for chlorophyll, leaf sugar and leaf starch we combined measurements made per unit leaf area and per unit leaf mass, for leaf area we combined measurements per leaf and per plant and for photosynthesis we combined measurements made at saturated and at experimental light conditions. Responses were measured at the end of the experiment and represent the status of the plant at that moment.

The freeware program DataThief III [55] was used to extract data from figures if the data was only reported in graphical form and could not be gathered from authors. In graphs where the error was not shown, as it was smaller than the symbol, the outmost margin of the symbol was taken as the error value. Most studies contributed more than one experiment to the database, e.g. various different N or [CO_2_] treatments.

Nutrient limitation is defined as the limitation of the productivity of a plant due to inadequate supply of the nutrient in the soil [56]. To examine N limitation in crops we need to compare a control, non-limiting N supply with an experimental, growth-limiting N supply. Given that it would not be possible to define a general non-limiting N supply across different crop species and across different experiments due to (1) the great variability in crop N demands between different crop species and at different crop development stages, as well as (2) the wide variety of experimental N application methods (eg in terms of the growth medium, the type of N input applied or the frequency of N application), the maximum N supply in each experiment was assumed to be the non-limiting control N supply and limiting N supplies were defined relative to this maximum rate (even when authors did not specify whether this maximum N supply was non-limiting). The *N limitation rate* was thus expressed relative to the control rate, ranging from 0% N limitation (i.e. control rate, maximum N supply) to 100% (maximum N limitation, i.e. zero N supply).

For some response variables—namely chlorophyll, Rubisco, leaf starch, and leaf sugar- we did not find enough data expressed per unit leaf area and per unit leaf mass to allow separate analyses for each unit (Table 1). For Rubisco, only one experiment per unit mass was included, and no formal testing of differences between units was performed. For chlorophyll, we also included studies reporting values measured with a SPAD device (a reliable proxy for leaf chlorophyll concentration, [57]) as we did not detect any consistent difference in the effects based on SPAD values (14% of the experiments) compared to area (73%) or mass-based (13%) values. For starch and sugars 12% and 26% of the experiments reported mass-based effects, respectively. For leaf area, instead, we combined measurements per leaf (21% of experiments) and per plant (79%), and for photosynthesis we combined measurements made at saturated (83%) and at experimental (10%) light conditions. Removing mass-based chlorophyll, leaf starch and leaf sugar measurements, or removing leaf-level leaf area data or photosynthesis measurements made at experimental light conditions had a negligible impact on the overall results but including these data allowed us to explore more species and test more explanatory variables.

For the investigated response variables, each experiment was standardized prior to analyses by expressing the effect of N limitation (Experimental N limited treatment, E) relative to the control (Control N treatment, C), i.e., the response ratio (*rr*). The log_e_ of the response ratio was used to linearize and improve normality of errors [58]. As a treatment and the control are independent in a factorial experimental design, the variance (Var) of log_e_ rr is calculated as (SD_E_^2^ ⁄ n_E_E^2^) + (SD_C_^2^ ⁄n_c_C^2^) [58], where n is the sample size. For convenience in the interpretation of our results we report values as percent change (i.e. [exp(log_e_ rr)-1] x 100) under N limitation, unless response ratios are compared to each other.

### Statistical analyses

Given that plant physiology predicts that N limitation influences plant responses, we are not interested in testing the existence of an effect (as often done in classical meta-analyses), but we are rather interested in testing and quantifying how various factors (e.g., N supply, N fixation, CO_2_ level) influence the effect of N limitation on our response variables. We therefore used a meta-regression, which is an extension of a classic meta-analysis and allows a mix of continuous (here N limitation) and categorical explanatory variables [59,60]. For all explanatory variables, we examined collinearity, as well as means and distribution of log_e_ rr and sample size among categories or along the range of continuous variables. As a first step of our meta-regression analyses, we analysed if explanatory variables related to the experimental setup (Table 2) could bias our results. Secondly, we tested the influence of experimental treatments and physiological crop characteristics (i.e. the variables of interest in this study, Table 2) on the response variables. Given that N limitation represents the focus variable in our study, we included it as a continuous variable in all models. As the N limitation effect could have a non-linear relationship to the explanatory variable *N limitation rate* (e.g. N limitation effect does not occur until severe N limitation is present), we also tested a quadratic term for this variable. Quadratic terms can result in odd model fits, although statistically significant, and models were also evaluated based on fit to the data (e.g. where the fitted line must pass through the origin, i.e. treatment and control are the same, and residuals should indicate equal variance).

**Table 2:** List and definition of explanatory variables and their classes.

Variable	Class	Definition
***Experimental treatments***		
N limitation rate	continuous (relative to control non-limiting N supply), 0–100%	
[CO_2_]	ambient; elevated	≤ 400 ppm; > 400 ppm
***Crop characteristics***		
Legume status	no; yes, no nodules; yes, with nodules	not a legume; legume but without N-fixing bacteria; legume and inoculated with N-fixing bacteria
Plant photosynthesis	C3; C4	
Plant group	monocotyl; dicotyl	
Crop type	cereals; fiber crops; pulses; oilseed crops; roots & tubers	see S1 Table for definitions
Crop species	wheat; maize; rice; barley; sorghum; cotton; soybean; rape-seed; common bean; potato; cassava	see S1 Table for definitions
***Experimental setup***		
N source	NO_3_^-^; NH_4_^+^; NO_3_^-^ and NH_4_^+^	
duration of N limitation	≤ 1/2; >1/2; entire	proportion of growth period in which N limitation was implemented
frequency N application	< 1; 1–2; 3–7; >7	more than 1x per day; every 1–2 days; every 3–7 days; less than 1x per week
pH control	yes; no	whether the pH of the growth medium was monitored and held constant
growth medium	soil	any type of soil (e.g. peat, loam, garden soil) or a mixture of soil with other substrates
	sand	sand without any other substrate; sandy soil is categorized as soil
	inert	growth on a solid, inert potting medium (e.g. arcillite, perlite, vermiculite)
	hydroponic	defined as growth in mineral nutrient solutions, with no solid medium for the roots
growth facility	growth chamber	crops grown in controlled environment growth chambers
	greenhouse	crops grown in greenhouses
	pots outside	crops grown in pots in the field
pot size	small; medium; big	0.3–2.4 l; 2.5–9 l; > 9 l
stress	none	no intentional stress component other than N stress
	stress	low water availability, high temperature, low light intensity, parasite infection, high ozone or salt stress

Ideally, all explanatory variables should be included in the statistical models. However, the large number of variables and categories within some variables (e.g., crop type) in comparison with the number of studies, made this an unfeasible option due to potential problems with overfitting and the risk of finding spurious results [61,62]. Instead, we fitted individual models for each explanatory variable but always included the variable *N limitation rate* as a covariate because (i) this was the key variable of interest in our study, and (ii) it was identified as the major explanatory variable for most response variables. For some data-rich models we also ran models with multiple variables of interest (e.g., legume status and CO_2_ level) to investigate the sensitivity of parameter estimates and confidence bounds.

Individual observations in a meta-analysis are often assumed to be independent. However, this is rarely true and in this study we had two main types of dependence: i) *sampling dependence*: effects from different treatments from the same study, e.g. from different [CO_2_] treatments or N supplies sharing the same control group, and ii) *study dependence*: same study includes many experiment with separate control groups but still share the same experimental setup (e.g. multiple cultivars). Here we accounted for these dependencies by employing mixed linear models [60], fitted in the *metafor* (ver 1.9–9) package in R [63]. In short, the model can be expressed as y = Xβ + δ + κ + ε, where y is the response vector of effect sizes (log_e_ rr); X is the design matrix of explanatory variables; β is a vector of parameters to represent the effects of different explanatory variables; δ and κ are identity matrices with σ^2^_study_ (study variance, accounting for study dependence) and σ^2^_exp_ (experiment variance, the between experiment variation and often called residual heterogeneity in meta-analysis) along the diagonal, respectively; and ε represents sampling error within each experiment. The known sampling variance-covariance matrix of ε consists of experiment-specific variances on the diagonal and covariances between related experiments, i.e., the sampling dependence, included as off-diagonal blocks [64] (see Limpens *et al*., [44,65] for details). Models were fitted with the maximum-likelihood estimator [66]. Statistical significance test of explanatory variables was performed with log-likelihood ratio tests (LRT), comparing the reduced model (removing the variable of interest) with the full model. Uncertainty of the effects (coefficients) were approximated as 1.96xSEs of the fixed effects, which is the standard output of the *metafor* package. As the sample size is rather high in our study, this is a good approximation of the confidence intervals (CIs). Effects and CIs were backtransformed to percentage scale to facilitate interpretation.

To explore to what extent two response variables respond similarly to N limitation we performed bivariate mixed linear models [60], only including studies reporting both response variables of interest. A bivariate model is an extension of the model described above where random effects have different variances for each response variable and are allowed to be correlated. This can be described as adding a 4x4 var-covariance matrix where the between-responses covariance is the off-diagonal component and the response variance is on the diagonal. To test if the correlation coefficient (*r*) between responses is significantly different from zero we conducted a LRT, comparing the bivariate model with a model where *r* is zero (i.e. no covariance component). However, this approach assumes that the correlation between the variables within a study is zero, and we therefore performed sensitivity analyses to investigate the influence of this assumption on the estimated correlation coefficients by imputing simulated within-study correlations (from -0.5 to 0.5 and back-computed to corresponding covariances) in our bivariate models, and thereafter comparing the obtained correlation coefficients between models with and without within-study correlation.

To examine bias in our data, we plotted effect size versus variance and number of replicates. General model checking was done using residual analyses and the impact of influential data points on parameter estimates. The latter was done by examining Cook’s distance and if influential experiments were detected we re-ran models without these data points as a sensitivity analysis.

### Data statement

Data and R-code to reproduce the results are deposited on Figshare at https://doi.org/10.6084/m9.figshare.9916649.v1.

## Results

### General response of leaf-level variables to N limitation

**Fig 2:**
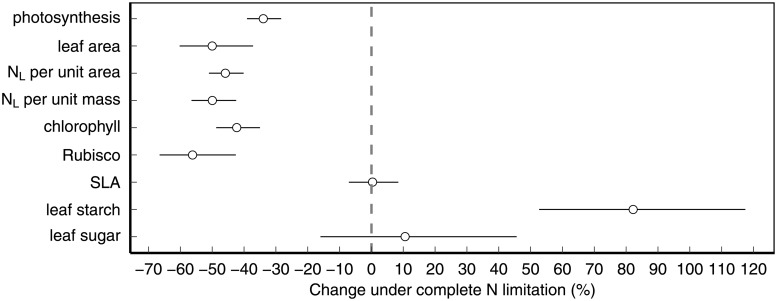
Effect of maximum N limitation on photosynthetic response variables. Estimated effects of experimental N limitation at *maximum* N limitation (i.e., zero nitrogen addition in the N limitation treatment) on photosynthetic response variables. Points show modelled estimates and whiskers represent 95% confidence interval. Sample sizes (number of studies, number of experiments) differ between variables: photosynthetic rate (50, 182), leaf area (21, 80), N_L_ per unit area (23, 120), N_L_ per unit mass (17, 95), chlorophyll content (32, 116), Rubisco content (13, 50), SLA (15, 68), leaf starch (8, 25), leaf sugars (12, 57). Leaf starch and leaf sugar includes measurements on both area and mass basis. Chlorophyll content represents mostly per unit area measurements but contains a small fraction of mass basis measurements (13%).

**Fig 3:**
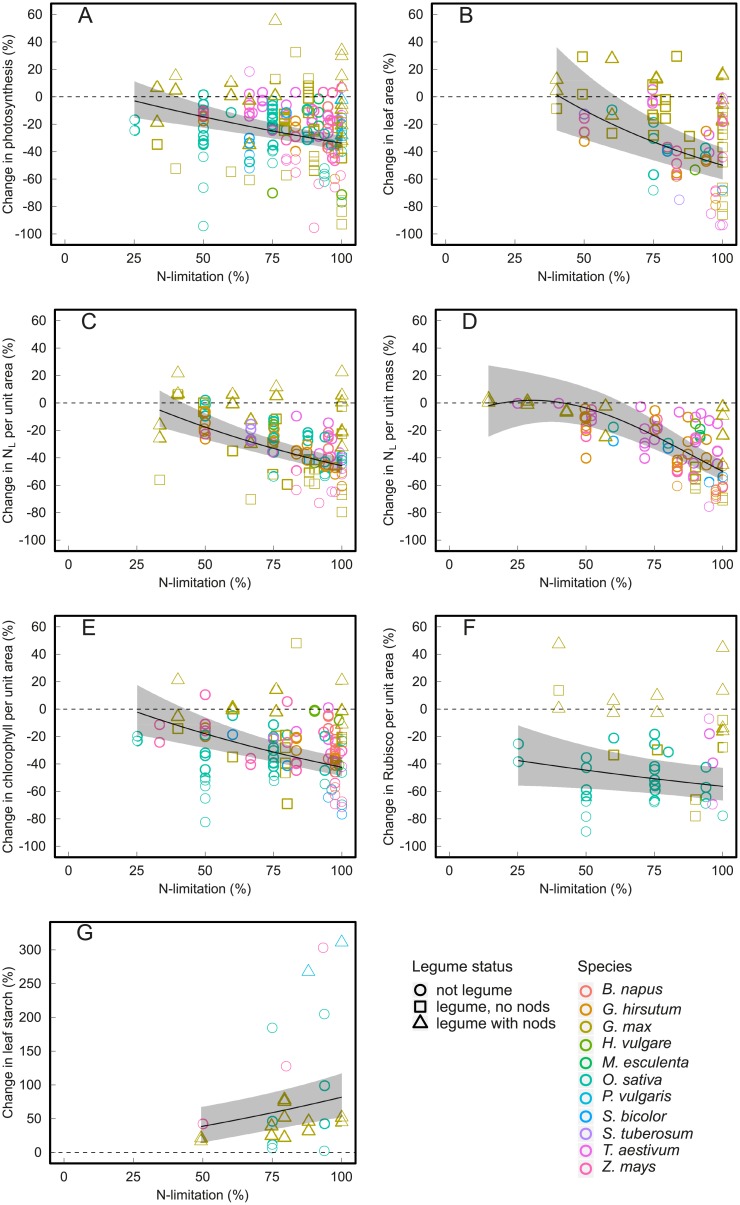
Effect of increasing N limitation on leaf-level variables. Relationship between leaf-level variables and experimental N limitation, where maximum N limitation (i.e., N limitation = 100%) means zero nitrogen addition in the N limitation treatment. Line shows modelled response (linear, except for N_L_ per unit mass, panel d, that also includes a quadratic term) and the shaded area indicates the 95% confidence interval. Each point represents one experiment. The individual panels show (a) photosynthesis (studies = 50, experiments = 182), (b) leaf area (21, 80), (c) N_L_ per unit area (23, 120), (d) N_L_ per unit mass (17, 95), (e) chlorophyll content (32, 116), and (f) Rubisco content (13, 50). See Table 3 for more details on model fit. We modelled a quadratic relationship for N_L_ per unit mass, as this model provided a better and more sensible fit to the data and was supported statistically (P<0.01, Table 3). A quadratic relationship was also weakly statistically supported for other variables (photosynthesis, leaf area, N_L_ per unit area; Table 3) but showed non-sensible fits driven by some data points at low N limitation and a linear fit was therefore preferred.

A total of 77 studies on 11 different crop species could be included in the meta-analysis (S3 Table), providing observations on 793 N limitation experiments. Most leaf-level variables examined here declined significantly under N limitation, with the exception of leaf starch content, which increased, and specific leaf area (SLA) and leaf sugar contents, which did not change significantly (Fig 2). The change in leaf-level variables increased with decreasing N supply (Table 3) and typically approached zero when experimental and control N supply were almost equal (Fig 3), indicating that the analysis is capturing N limitation well. It is, however, important to note that the modelled (typically linear) relationship between change in leaf-level variables and N limitation rate (Fig 3) typically only holds at intermediate to strong N limitation (e.g. >40% N limitation rate for leaf area, >33% for N_L_ per unit area, >25% for chlorophyll, Fig 3), due to the lack of data for low N limitation. An extrapolation of the linear relationship to low N limitation would result in positive impacts, which is not likely from a physiological perspective. Consequently, the relationship for these variables should be flat at these low N limitation rates for which we have no data.

**Table 3:** Results of the linear mixed models for all response variables with N limitation (linear or quadratic term) or CO_2_ level (ambient vs. elevated) as predictors.

Response	LRT	df	P	σ^2^_study_	σ^2^_exp_	N studies, N experiments	N ambient, N elevated
**Predictor N limitation, linear term**							
photosynthesis	20.71	1	**<0.001**	0.05	0.03	50, 182	/
leaf area	23.94	1	**<0.001**	0.22	0.09	21, 80	/
N_L_ per unit area	45.51	1	**<0.001**	0.04	0.04	23, 120	/
N_L_ per unit mass	63.35	1	**<0.001**	0.05	0.03	17, 95	/
chlorophyll	21.36	1	**<0.001**	0.07	0.05	32, 116	/
Rubisco	4.02	1	**<0.05**	0.18	0.06	13, 50	/
SLA	0.05	1	0.82	0.02	0.01	15, 68	/
leaf starch	14.34	1	**<0.001**	0.04	0.00	8, 25	/
leaf sugar	0.90	1	0.34	0.09	0.21	12, 57	/
**Predictor N limitation, quadratic term**							
photosynthesis	4.00	1	**<0.05**	0.05	0.03	50, 182	/
leaf area	3.38	1	0.07	0.22	0.09	21, 80	/
N_L_ per unit area	3.81	1	**0.05**	0.04	0.04	23, 120	/
N_L_ per unit mass	12.08	1	**0.001**	0.06	0.03	17, 95	/
chlorophyll	2.55	1	0.11	0.08	0.04	32, 116	/
Rubisco	0.68	1	0.41	0.18	0.06	13, 50	/
SLA	0.01	1	0.94	0.02	0.01	15, 68	/
leaf starch	0.11	1	0.74	0.04	0.00	8, 25	/
leaf sugar	3.00	1	0.08	0.12	0.19	12, 57	/
**Predictor [CO2]**							
photosynthesis	3.74	1	**0.05**	0.05	0.03	50, 182	157, 25
leaf area	0.69	1	0.41	0.23	0.09	21, 80	61, 19
N_L_ per unit area	1.86	1	0.17	0.03	0.04	23, 120	98, 22
N_L_ per unit mass	3.51	1	0.06	0.06	0.03	17, 95	58, 38
chlorophyll	1.53	1	0.22	0.07	0.05	32, 116	108, 8
Rubisco	2.37	1	0.12	0.16	0.06	13, 50	47, 3
SLA	0.12	1	0.73	0.01	0.01	15, 64	43, 21
leaf starch	0.08	1	0.77	0.04	0.00	8, 25	19, 6
leaf sugar	1.16	1	0.28	0.07	0.21	12, 57	43, 14

Models show the effect of experimental treatments (N limitation and growth CO_2_ level) on different photosynthetic response variables. LRT represents the log-likelihood ratio test, comparing the reduced model (removing the variable of interest) with the full model, and the P-value tests the statistical significance of the LRT. σ^2^_study_ represents the study variance, σ^2^_exp_ the experiment variance (also called the residual heterogeneity)–indicating the size and distribution of unexplained variation. N in the last two columns refers to sample size. P values <0.05 are highlighted in bold.

### Influence of experimental setup on the N limitation response

We examined the influence of several experimental variables (see Table 2) on the N limitation effect (S5 Table). The only experimental variable that appeared to show a somewhat robust effect was N source: crops receiving only NH_4_^+^ as N source showed a smaller N limitation effect on photosynthesis and leaf area than crops receiving only NO_3_^-^ as N source (S1 Fig). However, further sub-group analyses revealed that these effects were at least partly driven by individual crop species or individual studies, and more importantly, N source did not affect the general conclusions, i.e. our findings hold within N source groups (see Supplementary Discussion, S1 Text).

### Influence of crop characteristics on the N limitation response

**Fig 4:**
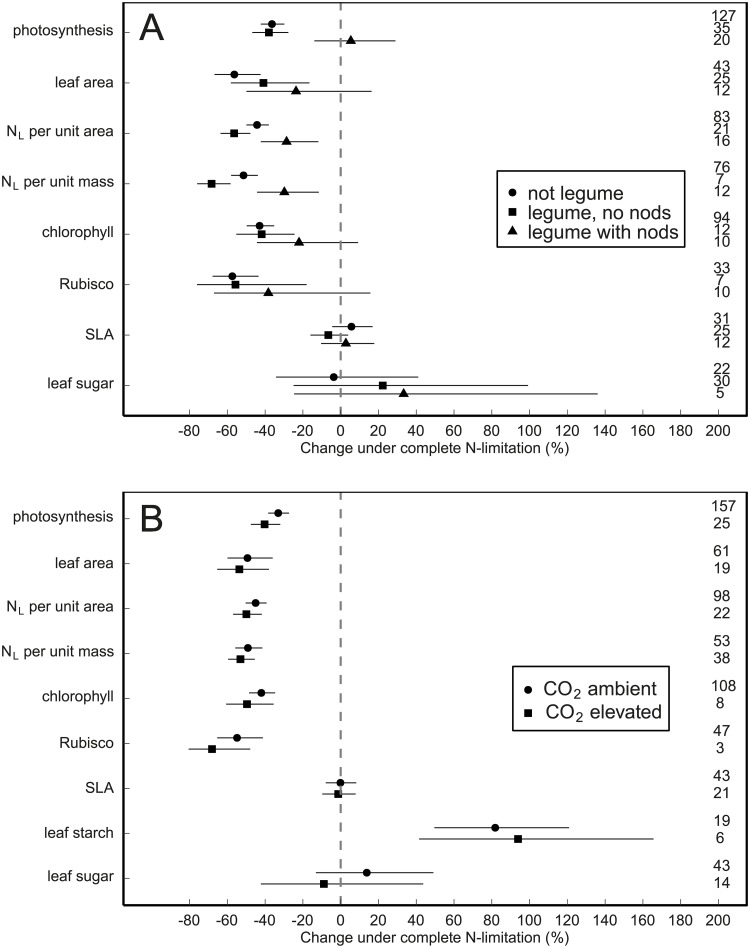
N limitation response of different legume status and CO_2_ experimental levels. N limitation response of crops (model estimate and 95% CI) with different legume status (panel a), and at different [CO2] levels (panel b). The factors of the variable legume status (panel a) are *not legume*, *legume*, *no nodules* and *legume*, *with nodules*. Numbers on the right in each panel indicate the number of experiments in each group. Leaf starch is not included in panel (a) as there are no studies with nodulating legumes.

There was some support that crop species differed in their response to N limitation for some variables (p = 0.05–0.1, S4 Table), but this crop species effect disappeared when legume status was included in the model (Results not shown). Differences between crop species could thus be explained primarily by difference between N-fixing and non-N-fixing crops: leguminous crops that were nodulating increased photosynthetic rate by 5% under N limitation, while legumes that were not nodulating decreased photosynthesis by 38% and non-leguminous crops reduced it by 36% (Fig 4a). A similar pattern was observed across all leaf-level variables examined, although not statistically significant for Rubisco (Table 4). No other crop trait showed any consistent influence on the N limitation response (S4 Table), as the observed C3/C4 effect on N_L_ was confounded by legume status, and the mono-/dicotyl effect on chlorophyll was confounded by N source (Results not shown).

**Table 4:** Results of the linear mixed models for all response variables with legume status as predictor.

Response	LRT	df	P	σ^2^_study_	σ^2^_exp_	N studies, N experiments	Factors (N)
photosynthesis	29.25	2	**<0.001**	0.06	0.03	50, 182	no (127), yes nod (20), yes non nod (35)
leaf area	5.46	2	0.07	0.20	0.09	21, 80	no (43), yes nod (12), yes non nod (25)
N_L_ per unit area	29.98	2	**<0.001**	0.03	0.03	23, 120	no (83), yes nod (16), yes non nod (21)
N_L_ per unit mass	33.82	2	**<0.001**	0.05	0.02	17, 95	no (76), yes nod (12), yes non nod (7)
chlorophyll	5.21	2	0.07	0.07	0.05	32, 116	no (94), yes nod (10), yes non nod (12)
Rubisco	4.56	2	0.10	0.16	0.06	13, 50	no (33), yes nod (10), yes non nod (7)
SLA	5.36	2	0.07	0.01	0.01	15, 68	no (31), yes nod (12), yes non nod (25)
leaf starch	/	/	**/**	/	/	8, 25	no (11), yes non nod (14)
leaf sugar	1.49	2	0.48	0.11	0.21	12, 57	no (22), yes nod (5), yes non nod (30)

Models show the effect of legume status on different photosynthetic response variables. The factors of the variable legume status are *no* (no legume), *yes nod* (legume, with nodules) and *yes no nod* (legume, no nodules). LRT represents the log-likelihood ratio test, comparing the reduced model (removing the variable of interest) with the full model, and the P-value tests the statistical significance of the LRT. σ^2^_study_ represents the study variance, σ^2^_exp_ the experiment variance (also called the residual heterogeneity)–indicating the size and distribution of unexplained variation. Note that the variable *N limitation rate* was included as a covariate in all models. N in the last two columns refers to sample size. P values <0.05 are highlighted in bold.

### N limitation response under elevated [CO_2_]

The photosynthetic rate showed a stronger response to N limitation under elevated than under ambient [CO_2_] levels (Table 3), decreasing by -40% under elevated, compared to -33% under ambient [CO_2_] at 100% N limitation (Fig 4b). While the N limitation response of other leaf-level variables–including leaf area, N_L_ per unit area and per unit mass, chlorophyll, Rubisco content, and leaf starch contents—was also slightly stronger under elevated [CO_2_] levels (Fig 4b), this difference was not statistically significant at P < 0.05 (Table 3).

### Relative changes of different leaf-level variables under N limitation

**Fig 5:**
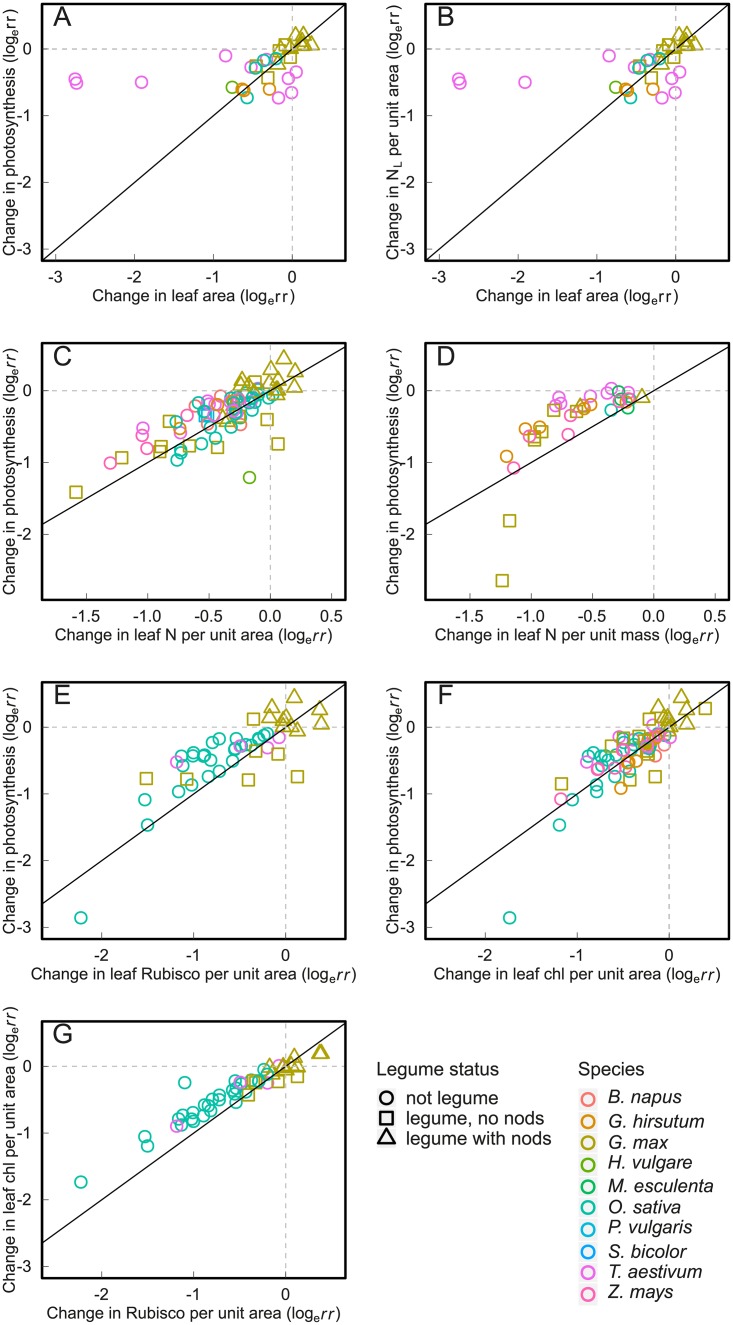
Correlation in the response of various leaf-level variables to N limitation. (a) photosynthetic versus leaf area responses, (b) leaf nitrogen (N_L_) per unit area versus leaf area responses, (c) photosynthetic versus N_L_ per unit area response, (d) photosynthetic versus N_L_ per unit mass response, (e) photosynthetic versus Rubisco content response, (f) photosynthetic versus chlorophyll response, (g) chlorophyll versus Rubisco response. Black line represents a 1:1 relationship. Each dot represents one experiment in which both responses were measured. Correlation coefficients: (a) *r* = 0.32, LRT = 2.6, P = 0.05, N = 53; (b) *r* = 0.42, LRT = 2.23, P = 0.07, N = 33; (c) *r* = 0.79, LRT = 17.6, P < 0.0001, N = 83; (d) *r* = 0.90, LRT = 6.0, P < 0.01, N = 34; (e) *r* = 0.91, LRT = 20.1, P < 0.0001, N = 48; (f) *r* = 0.87, LRT = 23.9, P < 0.0001, N = 78; (g) *r* = 0.99, LRT = 31.9, P < 0.0001, N = 48.

Across all studies and all crop species leaf area declined more strongly (-50%) than photosynthesis (-34%) under complete N limitation (i.e. zero N addition) compared to treatments with non-limiting N addition (Fig 2), and this was true for many individual experiments as well (Fig 5a; experiments above the 1:1 line). But different crop species and crop types differed slightly in this pattern: Most non-legume crops decreased both leaf area and photosynthesis, but decreased leaf area more strongly than photosynthesis. Wheat (*Triticum aestivum* L.), instead, maintained photosynthetic rate but decreased leaf area, however, this pattern was due to one single study (i.e., Evans 1983). Only soybean (*Glycine max* (L.) Merr.), if not enabled to form nodules, reduced its photosynthetic rate but did not show a strong decline in leaf area (Fig 5a).

Changes in photosynthetic rate and changes in N_L_ per unit area (r = 0.79, LRT = 17.6, P < 0.0001, N = 83), as well as per unit mass (r = 0.90, LRT = 6.0, P < 0.01, N = 34) were highly correlated, both within and between different crop species (Fig 5c and 5d). Typically, N_L_ per unit area (Fig 5c) and N_L_ per unit mass (Fig 5d) changed slightly more than leaf photosynthetic rate (i.e. more points falling above the 1:1 line).

The correlation between changes in photosynthetic rate and changes in chlorophyll content (r = 0.87, LRT = 23.9, P < 0.0001, N = 78) was also high, and the close clustering around the 1:1 line (Fig 5f) suggests that crops tend to reduce photosynthetic rate to the same degree as their chlorophyll content. The relationship between changes in photosynthetic rate and Rubisco content was also strong (r = 0.91, LRT = 20.1, P < 0.0001, N = 96), but most experiments showed larger changes in Rubisco content than in photosynthetic rate (Fig 5e), and, on average, Rubisco changed 20% more than photosynthesis (P < 0.001). Experiments measuring both chlorophyll and Rubisco content also showed a strong correlation between these two variables (r = 0.99, LRT = 31.99, P < 0.0001, N = 48) and a stronger change in Rubisco than in chlorophyll content (Fig 5g), with Rubisco content changing, on average, 19% more than chlorophyll content (P < 0.0001).

Sensitivity analyses of the bivariate models indicated that model estimates and P-values were not much affected by moderate within-study correlation (r = -0.5 to 0.5). Correlation coefficients changed less than ± 0.15 and P-values ± 0.01, but mostly the changes were negligible. However, there was one exception: the correlation coefficient between N_L_ per unit area and leaf area responses (Fig 5b) ranged between 0.17 and 0.61, with corresponding P-values of 0.28 and <0.01, suggesting that the model was sensitive to unknown within-study correlations and that the relationship between N_L_ per unit area and leaf area may be stronger or weaker depending on within-study correlations.

## Discussion

### Reduction in leaf area versus photosynthesis under N limitation

Under limiting N supply plants are faced with a joint optimization problem [10,11]: should they reduce N_L_ in order to optimize photosynthetic N use efficiency (which is highest at low N_L_), or should they maintain N_L_ rather constant at a given light environment in order to optimize light use efficiency and instead reduce their leaf area in response to the limiting N supply?

Our meta-analysis suggests that under severe N limitation crops do both. On the one hand, they do reduce N_L_ per unit leaf area, but on the other hand, they also reduce leaf area (Fig 2), typically to similar degrees (Fig 5b). But our analysis also shows that under N limitation most crops reduce leaf area and N_L_ more strongly than photosynthetic rate (Fig 5a and 5d). The only exception to this pattern are some non-nodulating leguminous soybeans, which decrease photosynthesis considerably more than leaf area (Fig 5a). Nodulating legumes, on the other hand, are able to fully compensate for N limitation through N fixation.

Our analysis detected no differences between mono- and dicotyledons or C3 and C4 species in the response of leaf area and photosynthesis to N limitation. This relative consistent pattern of relative leaf area to photosynthesis decline across many crop species (Fig 5a) does not support the hypothesis that mono- and dicotyledonous crops differ in their response strategy to N limitation [13,16]. It also does not support the hypothesis that different crop species differ considerably in their response strategy to N limitation [11]. In our analysis maize (*Zea mays* L.), sorghum (*Sorghum bicolor* (L.) Moench), rice (*Oryza sativa* L.) and cotton (*Gossypium hirsutum* L.) showed strikingly similar patterns in the reduction of leaf area and photosynthesis under N limitation across multiple studies. Note that some observations on wheat showed stronger reductions in leaf area than other crops (Fig 5a and 5b). But given that all these observations came from a single study [51], we cannot draw general conclusions on wheat. The non-leguminous crops examined in our study thus respond to N limitation first of all through a reduction in leaf area and N_L_ and only to a smaller degree through a reduction in photosynthetic rate per unit leaf area.

### Sink versus source limitation

The generally stronger reduction of leaf area compared to photosynthesis under N limitation observed in our study could potentially be explained by the exponential nature of plant growth that leads to a compounding effect of photosynthesis reduction on leaf area growth over time, or by carbohydrates produced being allocated to root rather than leaf biomass—which is a typical response of plants in order to increase nutrient uptake under N limitation [17,67,68]. But the concurrent observation that N limitation leads to an overall increase in leaf starch content (Fig 2) suggests yet another explanation: The accumulation of starch indicates that under N limitation crops continue to produce carbohydrates, and these carbohydrates then accumulate in the leaf, as they cannot be used further for growth, as leaf expansion, as well as the transport of sugars to sink tissues like roots is limited by the availability of amino acids [69]. In fact, the increase in starch in our meta-analysis (+82%) is directly proportional to the reduction in N_L_ (-45%), as (1–0.45) x (1+0.82) = 1.001. A reduction of N_L_ might thus lead to a proportional increase in leaf storage carbohydrates, as the carbohydrates produced by photosynthesis cannot be converted into new plant tissue. As a secondary response, photosynthesis is then down-regulated, as photosynthesis in source tissue is being controlled by carbohydrate demand in sink tissue through a mechanism of sugar repression [69,70]. Photosynthesis may thus not be limited by N directly but is down-regulated due to an N limitation of photosynthate utilization [71,72]. This mechanism of sink limitation of photosynthesis remains controversial, but is generally supported in the literature [10,69,70,73–75].

**Fig 6:**
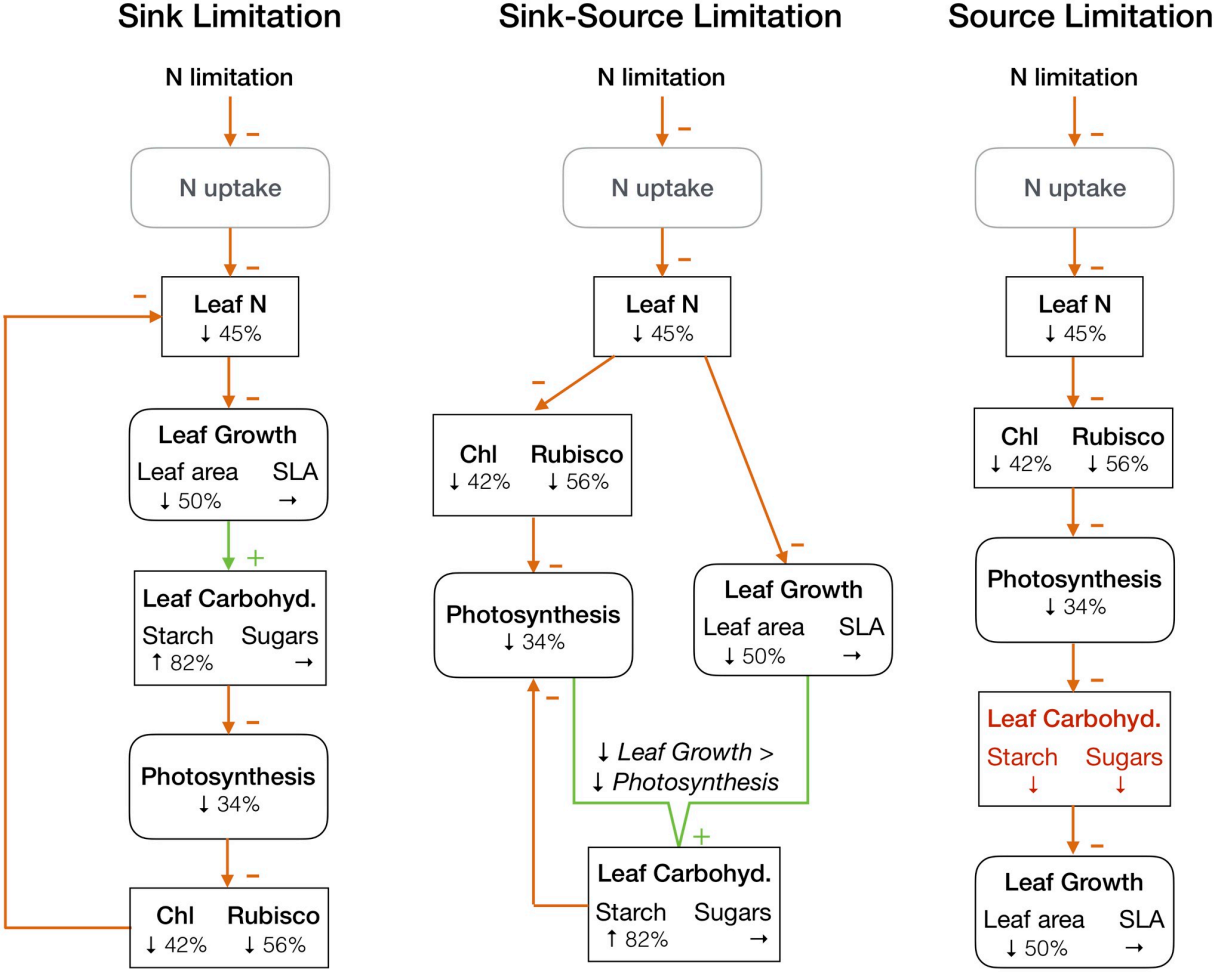
Potential relationships between photosynthesis and leaf growth of N-limited crops. In pure sink limitation (left panel), photosynthesis is limited by the use of carbohydrates in sink tissue. In pure source limitation (right panel), photosynthesis is limited by the availability of photosynthetic proteins. In combined sink and source limitation (middle panel), photosynthesis is limited by both the availability of photosynthetic proteins as well as the use of carbohydrates in sink tissue. Numbers and arrows show the results from this meta-analysis (at 100% N limitation). Square boxes show stocks, rounded boxes show processes. Arrows with a minus sign show negative impacts, arrows with a plus sign show positive impacts. Grey boxes indicate processes we did not examine in this meta-analysis. The pure source limitation (right panel) is not consistent with the results of our analysis, as under this mechanism we would expect a reduction in leaf carbohydrates.

Given the observed accumulation of starch, the results from our meta-analysis do not support a pure source limitation of crops under N limitation (Fig 6, right panel), but they are consistent, instead, with a mechanism of sink limitation (Fig 6, left panel), or possibly, a combined sink and source limitation (Fig 6, middle panel), as some level of direct reduction of photosynthetic proteins and thus photosynthetic rate most likely also takes place under severe N limitation [70].

### Influence of N limitation on leaf morphology

We did not observe an influence of N limitation on SLA of crops. Given that primary studies show differing responses of SLA to N limitation, this null result in our meta-analysis is not surprising. Some studies on crop species have observed decreases in SLA under N limitation due to increased accumulation of dry matter in the form of carbohydrates (particularly starch) and cell wall material [10,76], but other studies also observed no changes in SLA under N limitation [17,77], or even increases in SLA, for example due to re-allocation of carbohydrates to the reproductive organs under N stress [78] or due to decreases in leaf thickness under low N supply [79]. It could be that different crop species potentially show different responses in leaf morphology to N limitation [17], but we were not able to assess this due to rather small sample sizes for SLA (only 15 studies providing 68 observations).

### Influence of N limitation on the photosynthetic apparatus

We did not directly assess the proportion of N_L_ invested in Rubisco (which can be representative of the relative investment in the dark reactions of photosynthesis, i.e. the reactions of the Calvin cycle; [80]) versus chlorophyll (representative of the light reactions of photosynthesis, i.e. electron transport in the thylakoid membrane). But an analysis of studies measuring both changes in photosynthetic rate, N_L_, Rubisco and chlorophyll contents suggest that changes in N_L_, chlorophyll content, and photosynthetic rate are of similar magnitude under N limitation, while Rubisco content is reduced more strongly (Fig 5e–5g). The strong decline in Rubisco content under N limitation is probably caused by both the higher carboxylation efficiency at low N supply caused by lower CO_2_ resistance and higher CO_2_ diffusion to the carboxylation site under low leaf protein contents [81], and by Rubisco acting as a storage protein under high N supply [82,83]. The stronger decline in Rubisco versus chlorophyll content could also indicate photosynthetic acclimation to N limitation and decreased investment in the dark reactions of photosynthesis, but primary studies have typically not observed differences in *in vivo* electron transport versus Rubisco activity under low N supply [81,84]. Differently than the response to changing [CO_2_] or light environment, changing N supply does not appear to result in photosynthetic acclimation, or any changes in the relative rates of the dark or light reactions of photosynthesis [81,84].

### N limitation response under elevated [CO_2_]

In natural ecosystems the effect of CO_2_ fertilization is often limited by N availability [85]. Previous meta-analyses examining the effect of elevated [CO_2_] on photosynthesis also showed that the CO_2_ fertilization effect on photosynthesis was reduced under limiting N supply [35]. Our meta-analysis is in line with these findings, but we found a rather small effect of [CO_2_] compared to the overall N limitation effect. Nonetheless, our results confirm the increasing importance of N limitation under elevated [CO_2_], as the negative effect of N limitation on photosynthesis is greater under elevated [CO_2_] relative to ambient [CO_2_] (Fig 4b).

Under elevated [CO_2_] the carboxylation rate of Rubisco, and consequently the rate of C fixed per unit N in Rubisco is increased, and photosynthetic rate thus initially increases [86]. But if N supply is limiting, the additional carbohydrates produced cannot be incorporated into plant tissue due to the lack of sufficient N available for growth proteins, carbohydrates thus accumulate and photosynthesis is down-regulated by reducing the amount of Rubisco in the leaf [86]. Many primary studies [87–89], as well as a meta-analysis of the [CO_2_] fertilization effect [35] have thus also observed stronger decline of Rubisco content or activity in N-limited plants under elevated [CO_2_]. Our analysis supports these previous findings, although the statistical evidence is weak (P = 0.12), as only three studies out of 50 measured Rubisco content at elevated [CO_2_]. Similarly, while we did not detect an interaction of [CO_2_] level with N limitation on leaf sugar or leaf starch contents, this is probably caused by the few studies in our database that were conducted at elevated [CO_2_] (N = 6/25 of leaf starch and N = 14/57 of leaf sugar), which reduces our power to detect differences.

Overall, our analysis thus supports the hypothesis that the consideration of C-N feedbacks for the prediction of plant response becomes even more important under rising [CO_2_] [22,90].

### Implications for earth system modelling

Our analysis leads to five key conclusions on crop physiological processes under N limitation that are relevant for the depiction of C-N processes in TEMs:

1. N limitation impacts leaf growth more strongly than leaf photosynthesis.
2. Photosynthesis is sink-limited under N limitation.
3. N_L_ changes in response to N supply.
4. The fraction of N_L_ invested in Rubisco changes with N supply.
5. Leguminous crops respond differently to N limitation than other crops.

In the following we will discuss each of these conclusions in turn.

First, our analysis shows that crops respond to N limitation primarily through a reduction in leaf area rather than in photosynthesis and that the reduction in leaf area is not necessarily proportional to the reduction in photosynthesis (see Fig 5a). This is in line with the Nitrogen Nutrition Index framework [19], while in current global TEMs, N limitation often only influences leaf area through its influence on photosynthetic rate but does not have a direct impact on leaf growth (e.g. [22,24–26,91]; S6 Table). Our analysis suggests that the dependence of leaf growth on N availability needs to be represented more explicitly in TEMs.

Secondly, the leaf starch accumulation observed in our analysis shows that plant growth under N limitation is sink limited (Fig 6). Zaehle et al. ([30]) describe two common approaches for representing the influence of N limitation on plant growth in TEMs–the first approach uses an instantaneous down-regulation of photosynthesis under limiting N availability (e.g. [22,26,90,92]), the second approach estimates photosynthetic rate based on the linear relationship with N_L_, and N_L_ in turn is influenced by N uptake and availability (e.g. [27–29,91]). Both of these approaches represent N limitation primarily as a down-regulation of photosynthesis (also see S6 Table). The prevalence of sink limitation suggested by our study implies, however, that N limitation is mediated—as has also been suggested for other important physiological responses, e.g. to water stress [93,94], ozone stress [95] and acclimation to increased [CO_2_] [96]—by the source-sink feedback between photosynthate production and photosynthate utilization. Similarly as has been suggested for trees [97] and for vegetation modelling in general [20], the source-sink feedback could thus provide a useful framework for coupling water, C and nutrient dynamics of crops in TEMs. A better representation of sink limitation in TEMs may improve the representation of C-N feedbacks in TEMs, and potentially resolve current TEMs’ problems to replicate and explain experimental data, and accurately predict future ecosystem responses [30]. This will be especially relevant under elevated [CO_2_] conditions, where sink limitation of photosynthesis due to an exacerbated N limitation of growth might become even more important.

Thirdly, our analysis shows that crops optimize N_L_ for growth, not only based on the light environment within the canopy, but also based on N availability and that N_L_ changes in response to N supply [18]. This implies the need to estimate N_L_ dynamically as a function of N availability [23,27,29], instead of defining fixed N_L_ values or C:N ratios for certain plant or crop types, or only varying N_L_ depending on the light environment (as e.g. done by [22,24,90]; S6 Table).

Fourthly, our study also shows that Rubisco is reduced more strongly than other photosynthetic components under N limitation. This is most likely because Rubisco acts as N storage under high N levels, but also because at low leaf protein (and N_L_) concentrations a lower CO_2_ resistance increases the carboxylation efficiency of Rubisco. The correct depiction of CO_2_ mesophyll diffusion has been suggested as a general shortcoming in TEMs [98–100]. And many TEMs do not include a dependence of relative Rubisco content or carboxylation efficiency on N supply (e.g. [8,22,26]; S6 Table).

Fifthly, differently than sometimes hypothesized (e.g. [11,13]) we did not observe different response patterns to N limitation (neither in leaf area development versus photosynthesis, nor in allocation to different photosynthetic compounds) between different crop species or crop types—with the important exception of leguminous crops. This highlights the need to include N-fixing crops as a separate crop functional type in global TEMs and to depict biological N fixation in a process-based manner (which very few TEMs to date are doing; see S6 Table).

Note that our analysis focused on crops and it is not clear whether the observed relationships also apply to natural vegetation. But given that the crops included represent a range of different plant traits found in natural vegetation (S1 Table), across which the response patterns were similar, we venture that conclusions from our analysis should be transferable to natural herbaceous plant species.

### Knowledge gaps for plant physiology

The clear relationships observed between leaf-level variables and the level of N limitation–where the magnitude of change increased with increasing N limitation (Fig 3)—shows that, overall, our analysis captures N limitation well. This supports our approach of using a relative N limitation rate to handle varying degrees of N limitation in the experiments included in the meta-analysis. Yet study outcomes were still highly heterogeneous, even at similar N limitation rates. It is important to point out that this unexplained variability likely decreased the power to detect differences between subgroups with low sample sizes and these results should thus be interpreted with caution. A part of the unexplained variability can be attributed to potentially important experimental variables (e.g. crop development stage) that we could not analyse due to limited information provided in the experimental literature. Duration of N limitation (i.e. experimental time) did generally not show strong effects (S5 Table), but we hypothesize that duration and foremost poor control of N supply (which varies between studies) likely caused a substantial portion of the unexplained variability.

N experiments have been criticised for not providing sufficiently controlled conditions that allow a constant and defined N supply to plants [53,54]. As plants continuously take up N and thus reduce the available N supply in the medium, the N supply should ideally be continuously monitored and adjusted depending on plant N demand to achieve a constant plant N status [53,54,101]. As only 3% of experiments in our database applied N continuously or multiple times per day, our meta-analysis emphasizes the need for better-controlled and better-monitored crop N experiments. Moreover, semi-controlled experimental studies may not always be directly transferable to field conditions, as crops in the field often increase N supply through soil exploration by their roots [54]. Thus, field crops on farms probably often fall on the lower range of N limitation presented in our study. Better controlled field experiments are needed to investigate this.

We focused our study on the 15 globally most important crop species. However, due to a substantial crop and biome bias in experimental studies [102] we were not able to find data on several tropical (e.g. millet, sugarcane, oil palm), and temperate crop species (e.g. sugar beet) of global importance. Similarly, our meta-analysis revealed data gaps for some leaf-level variables like SLA or leaf sugar content, or Rubisco, chlorophyll or leaf starch content under elevated [CO_2_] conditions. More N limitation experiments should examine these under-studied crops and under-studied variables.

## Supporting information

S1 Table List of crop species included in the meta-analysis and their respective characteristics. (PDF)

S2 Table Prisma 2009 checklist. (PDF)

S3 Table List of studies included in the meta-analysis. (PDF)

S4 Table Results of the linear mixed models showing the effect of various crop characteristics on different leaf-level response variables. (PDF)

S5 Table Results of the linear mixed models testing the effect of experimental setup on different leaf-level response variables. (PDF)

S6 Table Overview of how N limitation is depicted in selected global Terrestrial Ecosystem Models (TEMs). (PDF)

S1 Fig N limitation response of crops with different N sources applied (ammonium-nitrate, nitrate, ammonium and urea). (PDF)

S1 Text Supplementary Discussion—Experimental variables. (PDF)
