# Supplementary material for: A meta-analysis of crop response patterns to nitrogen limitation for improved model representation
Source: PLoS One. 2019 Oct 17;14(10):e0223508. doi: 10.1371/journal.pone.0223508 (PMC6797162; doi:10.1371/journal.pone.0223508)
Supplement: S1 Table — (PDF) [file pone.0223508.s001.pdf]

S1 Table. List of crop species included in the meta-analysis and their respective characteristics. Included crop species represent the 10 worldwide most important crops in total area harvested and total amount produced in the year 2014, based on information from FAOSTAT (2017).

**Crop species**	**Crop type**	**C3/ C4**	**Leg-ume**	**Mono/ dicotyl**	**Included in analysis**
Wheat *(Triticum aestivum)*	cereal	C3	no	mono	yes
Maize *(Zea mays)*	cereal	C4	no	mono	yes
Rice *(Oryza sativa)*	cereal	C3	no	mono	yes
Barley *(Hordeum vulgare)*	cereal	C3	no	mono	yes
Sorghum *(Sorghum bicolor)*	cereal	C4	no	mono	yes
Millet *(Pennisetum glaucum)*	cereal	C4	no	mono	no
Cotton *(Gossypium hirsutum)*	fiber crop	C3	no	di	yes
Soybean *(Glycine max)*	oilseed and oleaginous fruits	C3	yes	di	yes
Rape *(Brassica napus)*	oilseed and oleaginous fruits	C3	no	di	yes
Oil palm *(Elaeis guineensis)*	oilseed and oleaginous fruits	C3	no	mono	no
Common bean *(Phaseolus vulgaris)*	pulses	C3	yes	di	yes
Sugarcane *(Saccharum officinarum)*	sugar crop	C4	no	mono	no
Sugar beet *(Beta vulgaris)*	sugar crop	C3	no	di	no
Potato *(Solanum tuberosum)*	edible roots and tubers	C3	no	di	yes
Cassava *(Manihot esculenta)*	edible roots and tubers	C3	no	di	yes
