# Supplementary material for: A meta-analysis of crop response patterns to nitrogen limitation for improved model representation
Source: PLoS One. 2019 Oct 17;14(10):e0223508. doi: 10.1371/journal.pone.0223508 (PMC6797162; doi:10.1371/journal.pone.0223508)
Supplement: S3 Table — (PDF) [file pone.0223508.s003.pdf]

S3 Table. List of studies included in the meta-analysis. LA- leaf area; SLA – specific leaf area; N_L – leaf N content; Chl – chlorophyll; Rub cont – Rubisco content; A – photosynthesis; Sug_L – leaf sugar; $Stch_L$ – leaf starch.

Reference	Variables	Crop species
Albert *et al.* [1]	Chl	rapeseed
Araya *et al.* [2]	N_L, Sug_L, $Stch_L$	common bean
Caputo & Barneix [3]	N_L, Sug_L	wheat
Cardoso-Vilhena & Barnes [4]	A, N_L	wheat
Cechin & Press [5]	A	rice
Cechin [6]	A	sorghum
Chapin *et al.* [7]	A, SLA	barley
Cramer & Lewis [8]	A	wheat, maize
Devienne *et al.* [9]	N_L	wheat
Echarte *et al.* [10]	A	maize
Erley et al. [11]	N_L, A	rapeseed
Evans [12]	LA, N_L, A, Chl	wheat
Fujikake *et al.* [13]	N_L	soybean
Gavito *et al.* [14]	A, SLA	wheat
Gleadow *et al.* [15]	A, N_L	cassava
Guitman *et al.* [16]	Sug_L, N_L, Chl	wheat
Heckathorn *et al.* [17]	A	maize
Henry *et al.* [18]	A	soybean
Hirasawa *et al.* [19]	N_L, A	rice
Hocking & Meyer [20]	N_L, SLA	wheat
Huang *et al.* [21]	Chl, Rub cont, A	rice
Imai *et al.* [22]	Chl, N_L, Rub cont	rice
Imai *et al.* [23]	Chl, N_L, Rub cont	rice
Kao & Forseth [24]	A	soybean
Khamis & Lamaze [25]	Sug_L, $Stch_L$, A, Chl	maize
Khamis *et al.* [26]	A	maize
Koeslin-Findeklee *et al.* [27]	Chl	rapeseed
Koeslin-Findeklee *et al.* [28]	Chl, A	rapeseed
Kumagai *et al.* [29]	Chl, Rub cont, A	rice
Kumagai *et al.* [30]	LA, A	rice
Kumagai *et al.* [31]	Chl, Rub cont, A	rice
Kumagai *et al.* [32]	Chl, Rub cont, A	rice
Li *et al.* [33]	LA, N_L, Rub cont, A, Chl	rice
Li *et al.* [34]	Chl, LA, Rub cont, A, N_L	wheat
Lu & Zhang [35]	Chl, A	maize
Luo *et al.* [36]	A, N_L, SLA	soybean
Maekawa & Kokobun [37]	A, N_L, Chl, Rub cont, LA, SLA	soybean
Maekawa *et al.* [38]	N_L, A	soybean
Makino & Osmond [39]	A, N_L	wheat
Makino *et al.* [40]	A, N_L	rice
Makino *et al.* [41]	A, N_L, LA, Chl, Rub cont, Sug_L, $Stch_L$	rice
Mortimer *et al.* [42]	A	common bean
Müller *et al.* [43]	A, N_L	barley
Naegle *et al.* [44]	LA	soybean

Nakamura *et al.* [45]	LA, N_L, A, SLA	soybean
Nakano *et al.* [46]	Sug_L, $Stch_L$, N_L, A, Chl	rice
Pons & Pearcy [47]	N_L, Chl, A	soybean
Prinsi *et al.* [48]	Chl, Sug_L	maize
Pugnaire & Chapin [49]	N_L, LA, SLA	barley
Robinson [50]	LA, Sug_L, $Stch_L$, A, Chl, SLA	soybean
Robinson [51]	LA, Sug_L, $Stch_L$, A, Chl, SLA	soybean
Robinson [52]	A, Chl, Sug_L, $Stch_L$	soybean
Rogers *et al.* [53]	N_L, LA, SLA	wheat
Rogers *et al.* [54]	LA, N_L	cotton
Schlemmer *et al.* [55]	Chl	maize
Simier *et al.* [56]	Chl, N_L	sorghum
Sims *et al.* [57]	LA	soybean
Sims *et al.* [58]	A, Sug_L, N_L, $Stch_L$, SLA	soybean
Sims *et al.* [59]	N_L, A, Rub cont	soybean
Sionit [60]	LA, SLA	soybean
Srivastava *et al.* [61]	N_L	soybean
Tian *et al.* [62]	A	maize
Tóth *et al.* [63]	A, LA	maize
Vos & Biemond [64]	LA, SLA	potato
Vos & van der Putten [65]	N_L, SLA	potato
Vos *et al.* [66]	A, N_L	maize
Wang *et al.* [67]	Chl	barley
Wong [68]	LA, A, Chl, N_L	cotton, maize
Wong [69]	N_L, SLA	cotton
Wong *et al.* [70]	Chl, N_L, A	cotton, maize
Yamamoto *et al.* [71]	Chl	rice
Yamori *et al.* [72]	N_L, Rub cont, Chl, A	wheat, rice
Yashima *et al.* [73]	N_L, Sug_L	soybean
Yin *et al.* [74]	A	wheat
Zhao *et al.* [75]	Chl, LA	maize
Zhao *et al.* [76]	LA, A	sorghum
Zhou *et al.* [77]	Chl, LA, A	soybean

Supplementary References

1. Albert B, Le Cahérec F, Niogret M-F, Faes P, Avice J-C, Leport L, et al. Nitrogen availability impacts oilseed rape (Brassica napus L.) plant water status and proline production efficiency under water-limited conditions. Planta. 2012;236: 659–676.
2. Araya T, Noguchi K, Terashima I. Effect of nitrogen nutrition on the carbohydrate repression of photosynthesis in leaves of Phaseolus vulgaris L. J Plant Res. 2010;123: 371–9. doi:10.1007/s10265-009-0279-8
3. Caputo C, Barneix AJ. Export of amino acids to the phloem in relation to N supply in wheat. Physiol Plant. 1997;101: 853–860. doi:10.1111/j.1399-3054.1997.tb01073.x
4. Cardoso-Vilhena J, Barnes J. Does nitrogen supply affect the response of wheat

(Triticum aestivum cv. Hanno) to the combination of elevated CO2 and O3? J Exp Bot. 2001;52: 1901–1911.

5. Cechin I, Press MC. Influence of nitrogen on growth and photosynthesis of a C3 cereal, Oryza sativa, infected with the root hemiparasite Striga hermonthica. J Exp Bot. 1994;45: 925–930.

6. Cechin I. Photosynthesis and chlorophyll fluorescence in two hybrids of sorghum under different nitrogen and water regimes. Photosynthetica. Springer; 1998;35: 233–240. Available: http://www.springerlink.com/index/N721R4223V12V430.pdf

7. Chapin FS, Walter CHS, Clarkson DT. Growth response of barley and tomato to nitrogen stress and its control by absisic acid, water relations and photosynthesis. Planta. 1988;173: 352–366.

8. Cramer MD, Lewis OAM. The influence of NO3- and NH4+ nutrition on the carbon and nitrogen partitioning characteristics of wheat (Triticum aestivum L .) and maize (Zea mays L .) plants. Plant Soil. 1993;154: 289–300.

9. Devienne F, Mary B, Lamaze T. Nitrate transport in intact wheat roots:II. Long-term effects of NO 3 − concentration in the nutrient solution on NO 3 − unidirectional fluxes and distribution within the tissues5. J Exp Bot. 1994;45: 677–684. doi:10.1093/jxb/45.5.677

10. Echarte L, Rothstein S, Tollenaar M. The Response of Leaf Photosynthesis and Dry Matter Accumulation to Nitrogen Supply in an Older and a Newer Maize Hybrid. Crop Sci. 2008;48: 656–665. doi:10.2135/cropsci2007.06.0366

11. Erley G, Wijaya K-A, Ulas A, Becker H, Wiesler F, Horst WJ. Leaf senescence and N uptake parameters as selection traits for nitrogen efficiency of oilseed rape cultivars. Physiol Plant. 2007;130: 519–531. doi:10.1111/j.1399-3054.2007.00921.x

12. Evans JR. Nitrogen and photosynthesis in the flag leaf of wheat (Triticum aestivum L.). Plant Physiol. Am Soc Plant Biol; 1983;72: 297–302. Available: http://www.plantphysiol.org/cgi/content/abstract/72/2/297

13. Fujikake H, Yashima H, Sato T, Ohtake N, Sueyoshi K, Ohyama T. Rapid and reversible nitrate inhibition of nodule growth and N2 fixation activity in soybean (Glycine max (L.) Merr.). Soil Sci Plant Nutr. 2002;48: 211–217.

14. Gavito ME, Curtis PS, Mikkelsen TN, Jakobsen I. Interactive effects of soil temperature, atmospheric carbon dioxide and soil N on root development, biomass and nutrient uptake of winter wheat during vegetative growth. J Exp Bot. 2001;52: 1913–23. Available: http://www.ncbi.nlm.nih.gov/pubmed/11520880

15. Gleadow RM, Evans JR, McCaffery S, Cavagnaro TR. Growth and nutritive value of cassava (Manihot esculenta Cranz.) are reduced when grown in elevated CO. Plant Biol. 2009;11: 76–82. doi:10.1111/j.1438-8677.2009.00238.x

16. Guitman MR, Arnozis PA, Barneix AJ. Effect of source-sink relations and nitrogen nutrition on senescence and N remobilization in the flag leaf of wheat. Physiol Plantarium. 1991;82: 278–284.

17. Heckathorn SA, Poeller GJ, Coleman JS, Hallberg RL. Nitrogen availability alters patterns of accumulation of heat stress-induced proteins in plants. Oecologia. 1996;105: 413–418.

18. Henry LT, Raper Jr CD, Rideout JW. Onset of and recovery from nitrogen stress during reproductive growth of soybean. Int J Plant Sci. The University of Chicago Press; 1992;153: 178–185. Available: http://www.jstor.org/stable/2995640

19. Hirasawa T, Ozawa S, Taylaran RD, Ookawa T. Varietal Differences in Photosynthetic Rates in Rice Plants, with Special Reference to the Nitrogen Content of Leaves. Plant Prod Sci. 2010;13: 53–57. doi:10.1626/pps.13.53

20. Hocking PJ, Meyer CP. Effects of CO, enrichment and nitrogen stress on growth and partitioning of dry matter and nitrogen in wheat and maize. Aust J Plant Physiol. 1991;18: 339–356.

21. Huang ZA, Jiang DA, Yang Y, Sun JW, Jin SH. Effects of nitrogen deficiency on gas exchange, chlorophyll fluorescence, and antioxidant enzymes in leaves of rice plants. Photosynthetica. 2004;42: 357–364.

22. Imai K, Suzuki Y, Makino A, Mae T. Effects of nitrogen nutrition on the relationships between the levels of rbcS and rbcL mRNAs and the amount of ribulose 1.5-bisphosphate carboxylase/oxygenase synthesized in the eighth leaves of rice from emergence through senescence. Plant, Cell Environ. 2005;28: 1589–1600. doi:10.1111/j.1365-3040.2005.01438.x

23. Imai K, Suzuki Y, Mae T, Makino A. Changes in the synthesis of Rubisco in rice leaves in relation to senescence and N influx. Ann Bot. 2007/10/30. 2008;101: 135–144. doi:10.1093/aob/mcm270

24. Kao W-Y, Forseth IN. The effects of nitrogen, light and water availability on tropic leaf movements in soybean (Glycine max). Plant, Cell Environ. 1991;14: 287–293. doi:10.1111/j.1365-3040.1991.tb01503.x

25. Khamis S, Lamaze T. Maximal biomass production can occur in corn (Zea mays) in the absence of NO3 accumulation in either leaves or roots. Physiol Plant. 1990;78: 388–394. doi:10.1111/j.1399-3054.1990.tb09053.x

26. Khamis S, Lamaze T, Farineau J. Effect of nitrate limitation on the photosynthetically active pools of aspartate and malate in maize, a NADP malic enzyme C4 plant. Physiol Plant. 1992;85: 223–229.

27. Koeslin-Findeklee F, Meyer A, Girke A, Beckmann K, Horst WJ. The superior nitrogen efficiency of winter oilseed rape (Brassica napus L.) hybrids is not related to delayed nitrogen starvation-induced leaf senescence. Plant Soil. 2014;384: 347–362.

28. Koeslin-Findeklee F, Rizi VS, Becker MA, Parra-Londono S, Arif M, Balazadeh S, et al. Transcriptomic analysis of nitrogen starvation-and cultivar-specific leaf senescence in winter oilseed rape (Brassica napus L.). Plant Sci. 2015;233: 174–185.

29. Kumagai E, Araki T, Kubota F. Effects of nitrogen supply restriction on gas

exchange and photosystem 2 function in flag leaves of a traditional low-yield cultivar and a recently improved high-yield cultivar of rice (Oryza sativa L.). Photosynthetica. 2007;45: 489–495.

30. Kumagai E, Araki T, Ueno O. Effect of nitrogen-deficiency on midday photoinhibition in flag leaves of different rice (Oryza sativa L.) cultivars. Photosynthetica. 2009;47: 241–246.

31. Kumagai E, Araki T, Kubota F. Characteristics of Gas Exchange and Chlorophyll Fluorescence during Senescence of Flag Leaf in Different Rice (Oryza sativa L.) Cultivars Grown under Nitrogen-Deficient Condition. Plant Prod Sci. 2009;12: 285–292. doi:10.1626/pps.12.285

32. Kumagai E, Araki T, Ueno O. Comparison of Susceptibility to Photoinhibition and Energy Partitioning of Absorbed Light in Photosystem II in Flag Leaves of Two Rice (Oryza sativa L.) Cultivars that Differ in Their Responses to Nitrogen-Deficiency. Plant Prod Sci. 2010;13: 11–20.

33. Li Y, Gao Y, Xu X, Shen Q, Guo S. Light-saturated photosynthetic rate in high-nitrogen rice (Oryza sativa L.) leaves is related to chloroplastic CO2 concentration. J Exp Bot. 2009;60: 2351–60. doi:10.1093/jxb/erp127

34. Li D, Tian M, Cai J, Jiang D, Cao W, Dai T. Effects of low nitrogen supply on relationships between photosynthesis and nitrogen status at different leaf position in wheat seedlings. Plant Growth Regul. 2013;70: 257–263.

35. Lu C, Zhang J. Photosynthetic CO2 assimilation, chlorophyll fluorescence and photoinhibition as affected by nitrogen deficiency in maize plants. Plant Sci. 2000;151: 135–143.

36. Luo Y, Sims DA, Griffin KL. Nonlinearity of photosynthetic responses to growth in rising atmospheric CO2: an experimental and modelling study. Glob Chang Biol. 1998;4: 173–183. doi:10.1046/j.1365-2486.1998.00116.x

37. Maekawa T, Kokubun M. Correlation of Leaf Nitrogen, Chlorophyll and Rubisco Contents with Photosynthesis in a Supernodulating Soybean Genotype Sakukei 4. Plant Prod Sci. 2005;8: 419–426. doi:10.1626/pps.8.419

38. Maekawa T, Takahashi M, Kokubun M. Responses of a supernodulating soybean genotype, Sakukei 4 to nitrogen fertilizer. Plant Prod Sci. 2003;6: 206–212.

39. Makino A, Osmond B. Effects of nitrogen nutrition on nitrogen partitioning between chloroplasts and mitochondria in pea and wheat. Plant Physiol. 1991;96: 355–362.

40. Makino A, Nakano H, Mae T. Responses of Ribulose-1,5-biphosphate carboxylase, cytochrome-f, and sucrose synthesis enzymes in rice leaves to leaf nitrogen and their relationships to photosynthesis. Plant Physiol. 1994;105: 173–179.

41. Makino A, Sato T, Nakano H, Mae T. Leaf photosynthesis, plant growth and nitrogen allocation in rice under different irradiances. Planta. 1997;203: 390–398. doi:10.1007/s004250050205

42. Mortimer PE, Pérez-Fernández MA, Valentine AJ. Arbuscular mycorrhizae affect the N and C economy of nodulated Phaseolus vulgaris (L.) during NH4+ nutrition. Soil Biol Biochem. 2009;41: 2115–2121. doi:10.1016/j.soilbio.2009.07.021

43. Müller J, Braune H, Diepenbrock W. Photosynthesis-stomatal conductance model LEAFC3-N: Specification for barley, generalised nitrogen relations, and aspects of model application. Funct Plant Biol. 2008;35: 797–810. doi:10.1071/FP08088

44. Naegle ER, Burton JW, Carter TE, Rufty TW. Influence of seed nitrogen content on seedling growth and recovery from nitrogen stress. Plant Soil. 2005;271: 329–340.

45. Nakamura T, Koike T, Lei T, Ohashi K, Shinano T, Tadano T. The effect of CO2 enrichment on the growth of nodulated and non-nodulated isogenic types of soybean raised under two nitrogen concentrations. Photosynthetica. 1999;37: 61–70.

46. Nakano H, Makino A, Mae T. The effect of elevated partial pressures of CO2 on the relationship between photosynthetic capacity and N content in rice leaves. Plant Physiol. 1997;115: 191–198.

47. Pons TL, Pearcy RW. Nitrogen reallocation and photosynthetic acclimation in response to partial shading in soybean plants. Physiol Plant. 1994;92: 636–644. doi:10.1111/j.1399-3054.1994.tb03034.x

48. Prinsi B, Negri AS, Pesaresi P, Cocucci M, Espen L. Evaluation of protein pattern changes in roots and leaves of Zea mays plants in response to nitrate availability by two-dimensional gel electrophoresis analysis. BMC Plant Biol. 2009/08/25. 2009;9: 113. doi:10.1186/1471-2229-9-113

49. Pugnaire F, Chapin FS. Environmental and physiological factors governing nutrient resorption efficiency in barley. Oecologia. Springer; 1992;90: 120–126. Available: http://www.springerlink.com/index/U616559742755688.pdf

50. Robinson JM. Leaflet photosynthesis rate and carbon metabolite accumulation patterns in nitrogen-limited, vegetative soybean plants. Photosynth Res. 1996;50: 133–148.

51. Robinson JM. Influence of daily photosynthetic photon flux density on foliar carbon metabolite levels in nitrogen-limited soybean plants. Int J Plant Sci. 1997;158: 32–43.

52. Robinson JM. The influence of elevated foliar carbohydrate levels on the ascorbate: dehydroascorbate redox ratios in nitrogen-limited spinach and soybean plants. Int J Plant Sci. 1997;158: 442–450.

53. Rogers G, Milham P, Gillings M, Conroy J. Sink Strength May Be the Key to Growth and Nitrogen Responses in N-Deficient Wheat at Elevated CO 2. Aust J Plant Physiol. 1996;23: 253. doi:10.1071/PP9960253

54. Rogers GS, Milham PJ, Thibaud MC, Conroy JP. Interactions between rising CO2 concentration and nitrogen supply in cotton. I. Growth and leaf nitrogen concentration. Aust J Plant Physiol. 1996;23: 19–125. Available:

http://www.scopus.com/inward/record.url?eid=2-s2.0-0030023401&partnerID=40&md5=4fc90b8fea6005f50c2d6961400caa80

55. Schlemmer MR, Shanahan JF, Schepers JS, Francis DD. Remotely measuring chlorophyll content in corn leaves with differing nitrogen levels and relative water content. Agron J. 2005;97: 106–112.

56. Simier P, Constant S, Degrande D, Moreau C, Robins RJ, Fer A, et al. Impact of nitrate supply in C and N assimilation in the parasitic plant Striga hermonthica (Del.) Benth (Scrophulariaceae) and its host Sorghum bicolor L . Plant, Cell Environ. 2006;29: 673–681. doi:10.1111/j.1365-3040.2005.01449.x

57. Sims D, Seemann JR, Luo Y. Elevated CO2 concentration has independent effects on expansion rates and thickness of soybean leaves across light and nitrogen gradients. J Exp Bot. 1998;49: 583–591. doi:10.1093/jexbot/49.320.583

58. Sims D a., Seemann JR, Luo Y. The significance of differences in the mechanisms of photosynthetic acclimation to light, nitrogen and CO2 for return on investment in leaves. Funct Ecol. 1998;12: 185–194. doi:10.1046/j.1365-2435.1998.00194.x

59. Sims D a., Luo Y, Seemann JR. Comparison of photosynthetic acclimation to elevated CO2 and limited nitrogen supply in soybean. Plant, Cell Environ. 1998;21: 945–952. doi:10.1046/j.1365-3040.1998.00334.x

60. Sionit N. Response of soybean to two levels of mineral nutrition in CO2-enriched atmosphere. Crop Sci. Crop Sci Soc America; 1983;23: 329. Available: http://crop.scijournals.org/cgi/content/abstract/23/2/329

61. Srivastava AC, Tiku AK, Pal M. Nitrogen and carbon partitioning in soybean under variable nitrogen supplies and acclimation to the prolonged action of elevated CO 2. Acta Physiol Plant. 2006;28: 181–188.

62. Tian Q, Chen F, Zhang F, Mi G. Genotypic Difference in Nitrogen Acquisition Ability in Maize Plants Is Related to the Coordination of Leaf and Root Growth. J Plant Nutr. 2006;29: 317–330. doi:10.1080/01904160500476905

63. Tóth VR, Mészáros I, Palmer SJ, Veres S, Précsényi I. Nitrogen deprivation induces changes in the leaf elongation zone of maize seedlings. Biol Plant. 2002;45: 241–247.

64. Vos J, Biemond H. Effects of Nitrogen on the Development and Growth of the Potato Plant. 1. Leaf Appearance, Expansion Growth, Life Spans of Leaves and Stem Branching. Ann Bot. Oxford University Press; 1992;70: 27–35. doi:10.1093/oxfordjournals.aob.a088435

65. Vos J, van der Putten PEL. Effect of nitrogen supply on leaf growth, leaf nitrogen economy and photosynthetic capacity in potato. F Crop Res. 1998;59: 63–72.

66. Vos J, van der Putten PEL, Birch CJ. Effect of nitrogen supply on leaf appearance, leaf growth, leaf nitrogen economy and photosynthetic capacity in maize (Zea mays L.). F Crop Res. 2005;93: 64–73. doi:10.1016/j.fcr.2004.09.013

67. Wang F, Wang X, Zhao C, Wang J, Li P, Dou Y, et al. Alternative pathway is involved in the tolerance of highland barley to the low-nitrogen stress by

maintaining the cellular redox homeostasis. Plant Cell Rep. 2016;35: 317–328.

68. Wong SC. Elevated atmospheric partial pressure of CO2 and plant growth. I. Interactions of nitrogen nutrition and photosynthetic capacity in C3 and C4 plants. Oecologia. 1979;44: 68–74.

69. Wong SC. Elevated Atmospheric Partial Pressure of CO2 and Plant Growth. II. Non-structural carbohydrate content in cotton plants and its effect on growth parameters. Photosynth Res. Springer; 1990;23: 171–180. Available: http://www.springerlink.com/index/KP2421618282787K.pdf

70. Wong S, Cowan IR, Farquhar GD. Leaf conductance in relation to rate of CO2 assimilation: I. Influence of nitrogen nutrition, phosphorus nutrition, photon flux density, and ambient partial pressure of CO2 during ontogeny. Plant Physiol. Am Soc Plant Biol; 1985;78: 821–825. Available: http://www.plantphysiol.org/cgi/content/abstract/78/4/821

71. Yamamoto A, Shim I, Fujihara S, Yoneyama T, Usui K. Effect of Difference in Nitrogen Media on Salt-Stress Response and Contents of Nitrogen Compounds in Rice Seedlings. Soil Sci Plant Nutr. 2004;50: 85–93. Available: http://ci.nii.ac.jp/naid/110001720266/

72. Yamori W, Nagai T, Makino A. The rate-limiting step for CO2 assimilation at different temperatures is influenced by the leaf nitrogen content in several C3 crop species. Plant Cell Environ. 2011;34: 764–777.

73. Yashima H, Fujikake H, Sato T, Ohtake N, Sueyoshi K, Ohyama T. Systemic and local effects of long-term application of nitrate on nodule growth and N2 fixation in soybean (Glycine max [L.] Merr.). Soil Sci Plant Nutr. 2003;49: 825–834.

74. Yin X, Struik PC, Romero P, Harbinson J, Evers JB, van der Putten PEL, et al. Using combined measurements of gas exchange and chlorophyll fluorescence to estimate parameters of a biochemical C photosynthesis model: a critical appraisal and a new integrated approach applied to leaves in a wheat (Triticum aestivum) canopy. Plant, Cell Environ. 2009;32: 448–64. doi:10.1111/j.1365-3040.2009.01934.x

75. Zhao D, Raja Reddy K, Kakani VG, Read JJ, Carter G a. Corn (Zea mays L.) growth, leaf pigment concentration, photosynthesis and leaf hyperspectral reflectance properties as affected by nitrogen supply. Plant Soil. 2003;257: 205–218. doi:10.1023/A:1026233732507

76. Zhao D, Reddy K, Kakani V, Reddy V. Nitrogen deficiency effects on plant growth, leaf photosynthesis, and hyperspectral reflectance properties of sorghum. Eur J Agron. 2005;22: 391–403. doi:10.1016/j.eja.2004.06.005

77. Zhou X-J, Liang Y, Chen H, Shen S-H, Jing Y-X. Effects of rhizobia inoculation and nitrogen fertilization on photosynthetic physiology of soybean. Photosynthetica. 2006;44: 530–535. doi:10.1007/s11099-006-0066-x
