# Supplementary material for: A meta-analysis of crop response patterns to nitrogen limitation for improved model representation
Source: PLoS One. 2019 Oct 17;14(10):e0223508. doi: 10.1371/journal.pone.0223508 (PMC6797162; doi:10.1371/journal.pone.0223508)
Supplement: S4 Table — (PDF) [file pone.0223508.s004.pdf]

S4 Table. Results of the linear mixed models showing the effect of various crop characteristics (see Table 2 for description of variables) on different leaf-level response variables. σ^2_{study} represents the study variance, σ^2_{exp} the experiment variance (also called the residual heterogeneity) – indicating the size and distribution of unexplained variation. Note that the variable *N limitation rate* was included as a covariate in all models.

**Response**	**LRT**	**df**	**P**	σ^2_{study}	σ^2_{exp}	**N studies, N exper.**	**Factors (N)**
**Crop species**							
photosynthesis	15.85	9	0.07	0.04	0.03	50, 182	B. napus (8), G. hirsutum (9), G. max (51), H. vulgare (2), M. esculenta (3), O. sativa (49), P. vulgaris (4), S. bicolor (6), T. aestivum (26), Z. mays (24)
leaf area	4.90	7	0.67	0.27	0.09	21, 80	G. hirsutum (9), G. max (37), H. vulgare (1), O. sativa (8), S. bicolor (2), S. tuberousm (2), T. aestivum (12), Z. mays (9)
N_L per unit area	10.40	8	0.24	0.04	0.04	23, 120	B. napus (6), G. hirsutum (24), G. max (35), H. vulgare (2), O. sativa (28), P. vulgaris (2), S. tuberosum (3), T. aestivum (15), Z. mays (5)
N_L per unit mass	4.05	7	0.77	0.08	0.03	17, 95	G. hirsutum (27), G. max (19), H. vulgare (1), M. esculenta (3), O. sativa (2), S. bicolor (4), T. aestivum (29), Z. mays (10)
chlorophyll	11.81	7	0.11	0.07	0.05	32, 116	B. napus (16), G. hirsutum (9), G. max (22), H. vulgare (4), O. sativa (32), S. bicolor (4), T. aestivum (9), Z. mays (20)
Rubisco	1.72	2	0.42	0.17	0.06	13, 50	G. max (17), O. sativa (29), T. aestivum (4)
SLA	10.63	5	0.06	0.02	0.01	15, 68	G. hirsutum (9), G. max (37), H. vulgare (2), S. tuberosum (4), T. aestivum (12), Z. mays (4)
leaf starch	8.88	3	**0.03**	0.01	0.00	8, 25	G. max (12), O. sativa (8), P. vulgaris (2), Z. mays (3)
leaf sugar	8.64	4	0.07	0.05	0.22	12, 57	G. max (31), O. sativa (8), P. vulgaris (4), T. aestivum (6), Z. mays (8)
**Crop type**							
photosynthesis	4.55	4	0.34	0.04	0.03	50, 182	cereal (107), fibre crop (9), pulses (4), oilseed (59), roots (3)
leaf area	3.82	3	0.28	0.18	0.09	21, 80	cereal (32), fibre crop (9), oilseed (37), roots (2)
N_L per unit area	0.54	4	0.97	0.03	0.05	23, 120	cereal (50), fibre crop (24), pulse (2), oilseed (41), roots (3)
N_L per unit mass	2.49	3	0.43	0.05	0.03	17, 95	cereal (46), fibre crop (27), oilseed (19), roots (3)
chlorophyll	5.92	2	**0.05**	0.06	0.04	32, 116	cereal (69), fibre crop (9), oilseed (38)
Rubisco	0.30	1	0.58	0.15	0.06	13, 50	cereal (33), oilseed (17)
SLA	3.23	3	0.36	0.01	0.01	15, 68	cereal (18), fibre crop (9), oilseed (37), roots (4)

leaf starch	7.40	2	**0.03**	0.01	0.00	8, 25	cereal (11), legume (2), oilseed (12)
leaf sugar	5.35	2	0.07	0.05	0.22	12, 57	cereal (22), legume (4), oilseed (31)
**Plant photosynthesis**							
photosynthesis	0.59	1	0.44	0.05	0.03	50, 182	C3 (153), C4 (29)
leaf area	0.82	1	0.37	0.23	0.09	21, 80	C3 (69), C4 (11)
N_L per unit area	6.49	1	**<0.05**	0.03	0.04	23, 120	C3 (115), C4 (5)
N_L per unit mass	0.01	1	0.93	0.06	0.03	17, 95	C3 (81), C4 (14)
chlorophyll	3.45	1	0.06	0.07	0.04	32, 116	C3 (93), C4 (23)
SLA	3.51	1	0.06	0.02	0.01	15, 68	C3 (64), C4 (4)
leaf starch	1.36	1	0.24	0.03	0.00	8, 25	C3 (22), C4 (3)
leaf sugar	0.07	1	0.79	0.11	0.21	12, 57	C3 (49), C4 (8)
**Plant group**							
photosynthesis	3.29	1	0.07	0.05	0.03	50, 182	dicotyl (75), monocotyl (107)
leaf area	0.01	1	0.92	0.23	0.09	21, 80	dicotyl (48), monocotyl (32)
N_L per unit area	0.01	1	0.93	0.04	0.04	23, 120	dicotyl (70), monocotyl (50)
N_L per unit mass	0.03	1	0.87	0.06	0.03	17, 95	dicotyl (49), monocotyl (46)
chlorophyll	5.89	1	**<0.05**	0.07	0.04	32, 116	dicotyl (47), monocotyl (69)
Rubisco	0.30	1	0.58	0.19	0.06	13, 50	dicotyl (17), monocotyl (33)
SLA	1.14	1	0.29	0.02	0.01	15, 68	dicotyl (50), monocotyl (18)
leaf starch	0.00	1	0.98	0.06	0.00	8, 25	dicotyl (14), monocotyl (11)
leaf sugar	1.37	1	0.24	0.09	0.21	12, 57	dicotyl (35), monocotyl (22)
