# Supplementary material for: A meta-analysis of crop response patterns to nitrogen limitation for improved model representation
Source: PLoS One. 2019 Oct 17;14(10):e0223508. doi: 10.1371/journal.pone.0223508 (PMC6797162; doi:10.1371/journal.pone.0223508)
Supplement: S5 Table — (PDF) [file pone.0223508.s005.pdf]

S5 Table. Results of the linear mixed models testing the effect of experimental setup (see Table 2 in main text for description of variables) on different leaf-level response variables. σ^2_{study} represents the study variance, σ^2_{exp} the experiment variance (also called the residual heterogeneity) – indicating the size and distribution of unexplained variation. Note that the variable *N limitation* was included as a covariate in all models.

**Response**	**LRT**	**df**	**P**	σ^2_{study}	σ^2_{exp}	**N studies, N exper.**	**Factors (N)**
**N source**							
photosynthesis	9.76	2	**<0.01**	0.03	0.03	48, 180	amm (54), nit (67), nit + amm (59)
leaf area	7.81	2	**0.02**	0.12	0.08	20, 78	amm (23), nit (31), nit + amm (24)
N_L per unit area	2.52	2	0.28	0.02	0.05	23, 120	amm (40), nit (36), nit + amm (44)
N_L per unit mass	0.50	3	0.92	0.06	0.03	16, 80	amm (4), nit (65), nit + amm (3), urea (8)
chlorophyll	1.63	2	0.44	0.06	0.05	30, 111	amm (21), nit (39), nit + amm (51)
Rubisco	3.66	2	0.16	0.10	0.06	12, 49	amm (17), nit (2), nit + amm (30)
SLA	2.25	2	0.33	0.01	0.01	14, 67	amm (19), nit (33), nit + amm (15)
leaf starch	0.00	1	1.00	0.02	0.00	8, 25	nit (13), nit + amm (12)
leaf sugar	1.37	1	0.24	0.06	0.21	12, 57	nit (42), nit + amm (15)
**Duration of N limitation**							
photosynthesis	2.84	2	0.24	0.04	0.04	45, 160	<1/2 (20), >1/2 (26), entire (114)
leaf area	0.11	2	0.95	0.20	0.09	19, 77	<1/2 (4), >1/2 (4), entire (69)
N_L per unit area	0.80	2	0.67	0.03	0.06	22, 104	<1/2 (16), >1/2 (5), entire (83)
N_L per unit mass	8.67	2	**0.01**	0.04	0.03	16, 80	<1/2 (4), >1/2 (2), entire (74)
chlorophyll	4.88	2	0.09	0.05	0.05	29, 110	<1/2 (22), >1/2 (26), entire (62)
Rubisco	3.00	2	0.22	0.11	0.06	11, 47	<1/2 (10), >1/2 (12), entire (25)
SLA	1.74	1	0.19	0.01	0.01	15, 68	<1/2 (1), entire (67)
leaf starch	7.40	2	**0.03**	0.00	0.00	8, 25	<1/2 (11), >1/2 (2), entire (12)
leaf sugar	3.99	2	0.14	0.00	0.24	12, 57	<1/2 (16), >1/2 (5), entire (36)
**Frequency of N application**							
photosynthesis	2.47	3	0.48	0.05	0.03	46, 173	<1 (5), 1to2 (57), 3to7 (50), >7 (61)
leaf area	0.70	3	0.87	0.21	0.09	18, 73	<1 (7), 1to2 (26), 3to7 (19), >7 (21)
N_L per unit area	1.70	2	0.43	0.03	0.05	23, 120	1to2 (31), 3to7 (44), >7 (45)
N_L per unit mass	3.64	2	0.16	0.05	0.02	15, 76	1to2 (58), 3to7 (10), >7 (8)
chlorophyll	3.12	3	0.37	0.07	0.05	29, 110	<1 (5), 1to2 (42), 3to7 (27), >7 (36)
Rubisco	0.06	2	0.97	0.16	0.06	13, 50	1to2 (4), 3to7 (17), >7 (29)
SLA	4.74	3	0.19	0.01	0.01	14, 64	<1 (2), 1to2 (35), 3to7 (8), >7 (19)
leaf starch	1.49	2	0.48	0.00	0.00	8, 25	>1 (3), 1to2 (14), 3to7 (8)
leaf sugar	4.38	2	0.11	0.03	0.20	11, 55	>1 (6), 1to2 (39), 3to7 (10)

Response	LRT	df	P	σ^2_{study}	σ^2_{exp}	N studies, N exper.	Factors (N)
**Growth medium**							
photosynthesis	4.66	3	0.20	0.04	0.03	49, 179	hydroponic (52), inert (8), sand (28), soil (91)
leaf area	1.49	3	0.69	0.19	0.09	21, 80	hydroponic (11), inert (87, sand (8), soil (54)
N_L per unit area	2.65	3	0.45	0.03	0.05	23, 120	hydroponic (25), inert (3), sand (3), soil (89)
N_L per unit mass	19.63	3	**<0.001**	0.01	0.03	15, 77	hydroponic (10), inert (2), sand (29), soil (36)
chlorophyll	0.78	3	0.85	0.07	0.05	32, 116	hydroponic (53), inert (6), sand (11), soil (46)
Rubisco	0.15	1	0.70	0.16	0.06	13, 50	hydroponic (31), soil (19)
SLA	7.19	3	0.07	0.01	0.01	15, 68	hydroponic (1), inert (6), sand (13), soil (48)
leaf starch	3.88	3	0.28	0.00	0.00	8, 25	hydroponic (8), inert (6), sand (3), soil (8)
leaf sugar	6.30	4	0.18	0.00	0.22	12, 57	hydroponic (10), inert (11), sand (11), soil (25)
**pH control**							
photosynthesis	1.77	1	0.18	0.04	0.03	50, 182	no (143), yes (39)
leaf area	0.01	1	0.94	0.21	0.09	21, 80	no (70), yes (10)
N_L per unit area	0.51	1	0.48	0.03	0.05	23, 120	no (112), yes (8)
N_L per unit mass	0.01	1	0.91	0.06	0.03	16, 80	no (78), yes (2)
chlorophyll	0.04	1	0.84	0.07	0.05	31, 115	no (78), yes (37)
Rubisco	0.31	1	0.58	0.15	0.06	13, 50	no (27), yes (23)
SLA	3.22	1	0.07	0.01	0.01	15, 68	no (65), yes (3)
leaf starch	2.15	1	0.14	0.00	0.00	8, 25	no (14), yes (11)
leaf sugar	0.28	1	0.60	0.07	0.20	11, 55	no (39), yes (16)
**Growth facility**							
photosynthesis	2.37	2	0.31	0.04	0.03	49, 181	greenhouse (65), growth chamber (65), pots outside (51)
leaf area	1.81	2	0.40	0.19	0.09	21, 80	greenhouse (33), growth chamber (28), pots outside (19)
N_L per unit area	2.46	2	0.29	0.02	0.05	23, 120	greenhouse (53), growth chamber (27), pots outside (40)
N_L per unit mass	0.42	2	0.81	0.06	0.03	16, 80	greenhouse (48), growth chamber (24), pots outside (8)
chlorophyll	0.55	2	0.76	0.07	0.05	31, 115	greenhouse (73), growth chamber (24), pots outside (18)
Rubisco	2.82	2	0.24	0.12	0.06	13, 50	greenhouse (22), growth chamber (11), pots outside (17)
SLA	3.87	2	0.15	0.01	0.01	13, 66	greenhouse (26), growth chamber (25), pots outside (15)
leaf starch	5.08	1	**0.02**	0.00	0.00	8, 25	greenhouse (8), growth chamber (17)
leaf sugar	2.11	1	0.15	0.06	0.20	11, 55	greenhouse (19), growth chamber (36)

Response	LRT	df	P	σ^2_{study}	σ^2_{exp}	N studies, N exper.	Factors (N)
**Pot size**							
photosynthesis	1.61	2	0.45	0.05	0.04	40, 144	big (29), medium (74), small (41)
leaf area	4.58	2	0.10	0.16	0.09	19, 75	big (11), medium (40), small (24)
N_L per unit area	0.89	2	0.64	0.03	0.05	18, 92	big (10), medium (57), small (25)
N_L per unit mass	3.84	2	0.15	0.04	0.04	13, 61	big (7), medium (43), small (11)
chlorophyll	7.59	2	**0.02**	0.05	0.04	27, 100	big (20), medium (50), small (30)
Rubisco	4.00	2	0.14	0.13	0.04	11, 42	big (14), medium (13), small (15)
SLA	2.87	2	0.24	0.01	0.01	14, 56	big (4), medium (25), small (27)
leaf starch	0.03	1	0.86	0.00	0.00	6, 19	medium (13), small (6)
leaf sugar	5.11	1	**0.02**	0.01	0.21	10, 51	medium (32), small (19)
