# Supplementary material for: A meta-analysis of crop response patterns to nitrogen limitation for improved model representation
Source: PLoS One. 2019 Oct 17;14(10):e0223508. doi: 10.1371/journal.pone.0223508 (PMC6797162; doi:10.1371/journal.pone.0223508)
Supplement: S6 Table — (PDF) [file pone.0223508.s006.pdf]

S6 Table. Overview of how N limitation is depicted in selected global Terrestrial Ecosystem Models (TEMs), indicating whether crops are included ('Crops'), how N limitation is represented in the models ('N lim.'), whether N supply influences leaf area independently from its influence on NPP ('LAI N-dep.'), whether photosynthesis is sink limited, i.e. influenced by carbohydrate demand in sink tissue ('Sink lim.'), whether N_L is dependent on N supply ('N_L N-dep.'), whether the fraction of N_L invested in Rubisco content changes with N supply ('Rubisco N-dep.') and whether BNF is represented in the model ('BNF'). Models that do not depict the relationship discussed (as they, for example, do not include a representation of Rubisco) are indicated with a slash. Abbreviations used: LAI – Leaf Area Index; NPP – Net Primary Productivity; GPP – Gross Primary Productivity; BNF – Biological N Fixation; N_L – leaf N content.

**Model**	**Reference**	**Crops**	**N lim.**	**LAI N-dep.**	**Sink lim.**	**N_L N-dep.**	**Rubisco N-dep.**	**BNF**	**Details**
NCIM	Esser *et al.* [78]	no	NPP	no	no	yes	/	yes	NPP scaled based on N status of leaves
ISAM-NC	Jain *et al.* [79]	no	NPP	no	no	no	/	(yes)[1]	NPP scaled based on N status of plant
CN-TEM	Sokolov *et al.* [80], McGuire *et al.* [81]	no	NPP	no	no	no	/	no	NPP scaled based on plant C:N ratio & N supply
CLM4.5	Thornton *et al.* [82], Ghimire *et al.* [83]	no	NPP	no	yes	no	no	(yes)[1]	NPP scaled based on plant C:N ratio & N supply; feedback where N lim. leads to excess C that cannot be used for growth, which influences C:N ratio & down-regulates NPP
ORCHIDEE -CN	Zaehle & Friend [84]	no	NPP & LAI	yes	no	yes	yes[2]	(yes)[1]	N supply determines N_L and thereby NPP but also growth & allocation to leaves vs roots
LPJ-GUESS	Smith *et al.* [85], Olin *et al.* [86]	yes	NPP & LAI	yes	no	yes	no	(yes)[1]	N supply influences N_L and in turn NPP; reduced NPP due to N stress increases investment in root (& thus decreases LAI)
DayCent	Stehfest *et al.* [87], Parton *et al.* [88]	yes	biomass	yes	/	yes	/	(yes)[1]	N supply influences C:N ratio & minimum C:N ratio imposes constraint on new biomass produced under N limitation
LPJmL	von Bloh *et al.* [89], Bondeau *et al.* [90]	yes	NPP & LAI	yes	yes[3]	yes	yes	(yes)[1]	N supply influences N_L and in turn GPP and respiration; N stress increases investment in root, daily LAI increment is reduced under N stress
PEGASUS	Deryng *et al.* [91]	yes	NPP	no	no	/	/	(yes)[1]	empirical nutrient stress factor used to scale NPP

[1]not process-based; [2]but also chlorophyll content; [3]trees only
