# Supplementary material for: A meta-analysis of crop response patterns to nitrogen limitation for improved model representation
Source: PLoS One. 2019 Oct 17;14(10):e0223508. doi: 10.1371/journal.pone.0223508 (PMC6797162; doi:10.1371/journal.pone.0223508)
Supplement: S1 Fig — (PDF) [file pone.0223508.s007.pdf]

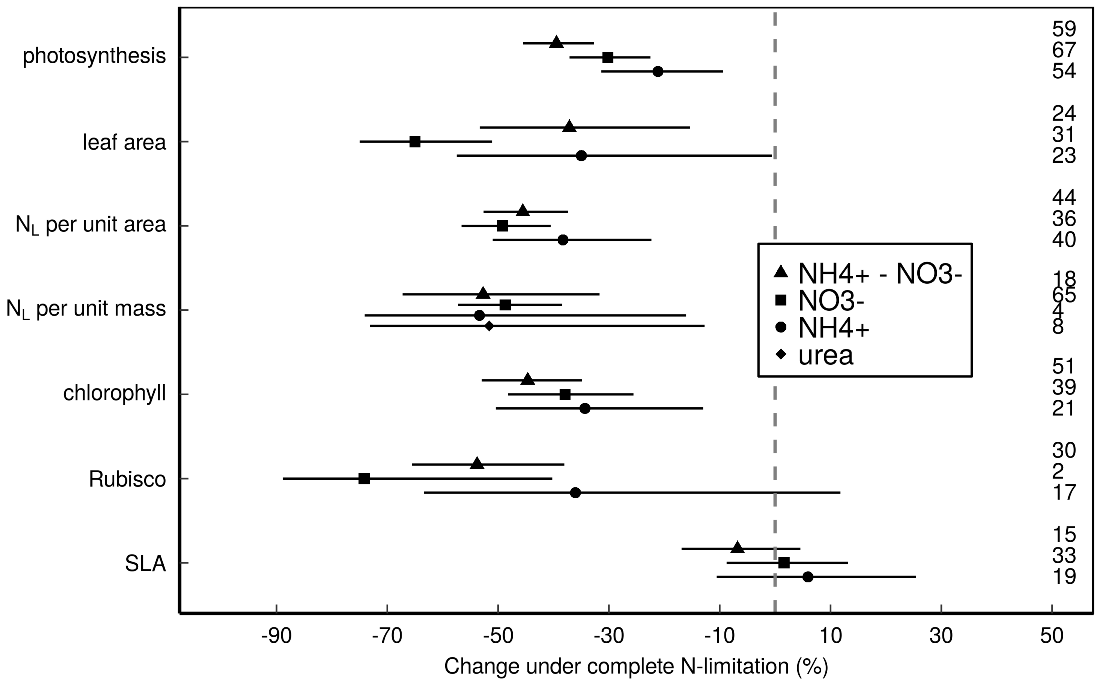

S1 Figure. N limitation response of crops (model estimate and 95% CI) with different N sources applied (ammonium-nitrate, nitrate, ammonium and urea). Numbers on the right indicate the number of experiments in each group. Leaf sugar and starch not shown as they did not have experiments with solely ammonium.
