# Supplementary material for: A meta-analysis of crop response patterns to nitrogen limitation for improved model representation
Source: PLoS One. 2019 Oct 17;14(10):e0223508. doi: 10.1371/journal.pone.0223508 (PMC6797162; doi:10.1371/journal.pone.0223508)
Supplement: S1 Text — (PDF) [file pone.0223508.s008.pdf]

S1 Text. Supplementary Discussion - experimental variables

We detected few effects of experimental variables ($p<0.05$, Table S4) on the response variables and these effects were in general of stochastic nature. For example, few response variables exhibited similar patterns and correlated response variables (see main text) could show different responses to experimental variables. Effects were often associated with a low sample size in one experimental group. Hence, the effect of the experimental factor was largely driven by one study, making the results equivocal (e.g. the significant results for N_L per unit mass).

The only slightly more robust results were the effects of N source on photosynthesis and leaf area. However, also these results were challenging to interpret. For photosynthesis, N limitation was less pronounced when compared to NH_4^+ addition, than for NO_3^- or NH_4^+ and NO_3^- combined (NH_4^+: -21%, NO_3^-: -30%, NH_4^+-NO_3^-: -39%, Fig. S1). These differences are not large and are driven by experiments examining legumes with nodes only using NH_4^+ as fertilizer. Consequently, N source had no effect if nod-producing legumes were excluded from the analysis. In contrast to photosynthesis, the N limitation effect on leaf area was stronger in experiments using NO_3^- as N supply (NH_4^+: -35%, NO_3^-: -65%, NH_4^+-NO_3^-: -37%, Fig. S1). As for photosynthesis, this effect of NO_3^- was less supported if nod-producing legumes were excluded from the analysis ($p=0.07$). Furthermore, one study had a major impact on these results [1, N=4] and the NO_3^- effect was strongly reduced and non-significant if this single study was excluded ($p=0.14$). It cannot be ruled out that NO_3^- exacerbated the N limitation effect, which could potentially be attributed to increased growth of plants receiving NO_3^- under non-limited conditions, as plants often take up NO_3^- more readily than NH_4^+ [e.g. 2], while NH_4^+ (especially when used as sole N source) can sometimes lead to toxicity symptoms [3] and the effect of N limitation might thus be smaller due to reduced growth of control plants receiving high NH_4^+ rates. But results were inconsistent among response variables, and importantly, our general conclusions hold even if NO_3^- experiments are excluded. For example, leaf area decreased significantly more than photosynthesis (Fig. 6a, main text) also when excluding NO_3^- experiments. To conclude, we acknowledge that N source may modify the outcomes of N limitation studies but to what extent remains uncertain. However, we did not find support that N source has clear consistent effects across measured responses and that N source affect our general conclusions.

Supplementary References

1. Evans JR. Nitrogen and photosynthesis in the flag leaf of wheat (Triticum aestivum L.). Plant Physiol. Am Soc Plant Biol; 1983;72: 297–302. Available: http://www.plantphysiol.org/cgi/content/abstract/72/2/297
2. Recous S, Machet J-M, Mary B. The fate of labelled ^{15}N urea and ammonium nitrate applied to a winter wheat crop: II. Plant uptake and N efficiency. Plant Soil. 1988;112: 215–224.
3. Britto DT, Kronzucker HJ. Review NH 4 + toxicity in higher plants: a critical review. Ecology. 2002;584.
